# The connection of α- and β-domains in mammalian metallothionein-2 differentiates Zn(II) binding affinities, affects folding, and determines zinc buffering properties

**DOI:** 10.1093/mtomcs/mfad029

**Published:** 2023-05-05

**Authors:** Avinash Kumar Singh, Adam Pomorski, Sylwia Wu, Manuel D Peris-Díaz, Hanna Czepczyńska-Krężel, Artur Krężel

**Affiliations:** Department of Chemical Biology, Faculty of Biotechnology, University of Wrocław, Joliot-Curie 14a, 50-383 Wrocław, Poland; Department of Chemical Biology, Faculty of Biotechnology, University of Wrocław, Joliot-Curie 14a, 50-383 Wrocław, Poland; Department of Chemical Biology, Faculty of Biotechnology, University of Wrocław, Joliot-Curie 14a, 50-383 Wrocław, Poland; Department of Chemical Biology, Faculty of Biotechnology, University of Wrocław, Joliot-Curie 14a, 50-383 Wrocław, Poland; Department of Chemical Biology, Faculty of Biotechnology, University of Wrocław, Joliot-Curie 14a, 50-383 Wrocław, Poland; Department of Chemical Biology, Faculty of Biotechnology, University of Wrocław, Joliot-Curie 14a, 50-383 Wrocław, Poland

**Keywords:** free zinc, metal affinity, protein–protein interaction, zinc probe, protein folding, interdomain interaction

## Abstract

Mammalian metallothioneins (MTs) are small Cys-rich proteins involved in Zn(II) and Cu(I) homeostasis. They bind seven Zn(II) ions in two distinct β- and α-domains, forming Zn_3_Cys_9_ and Zn_4_Cys_11_ clusters, respectively. After six decades of research, their role in cellular buffering of Zn(II) ions has begun to be understood recently. This is because of different affinities of bound ions and the proteins’ coexistence in variously Zn(II)-loaded Zn_4-7_MT species in the cell. To date, it has remained unclear how these mechanisms of action occur and how the affinities are differentiated despite the Zn(S-Cys)_4_ coordination environment being the same. Here, we dissect the molecular basis of these phenomena by using several MT2 mutants, hybrid protein, and isolated domains. Through a combination of spectroscopic and stability studies, thiol(ate) reactivity, and steered molecular dynamics, we demonstrate that both protein folding and thermodynamics of Zn(II) ion (un)binding significantly differ between isolated domains and the whole protein. Close proximity reduces the degrees of freedom of separated domains, making them less dynamic. It is caused by the formation of intra- and interdomain electrostatic interactions. The energetic consequence of domains connection has a critical impact on the role of MTs in the cellular environment, where they function not only as a zinc sponge but also as a zinc buffering system keeping free Zn(II) in the right concentrations. Any change of that subtle system affects the folding mechanism, zinc site stabilities, and cellular zinc buffer components.

## Graphical Abstract

**Graphical Abstract:**
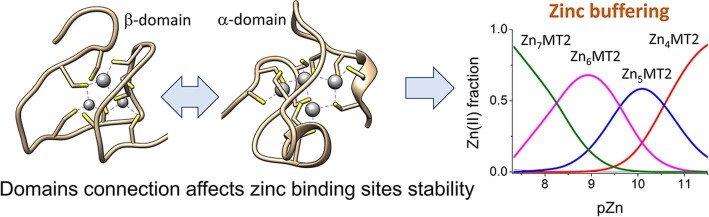
The close proximity of MT domains has a critical impact on the differentiation stability of zinc binding sites, their metalation mechanism, and their reactivity. It impacts the role of MTs in the cellular environment, where they function not only as a sponge of zinc but also as a buffering system of Zn(II) ions, keeping them in the right concentrations.

## Introduction

Mammalian metallothioneins (MTs) are a group of low molecular weight (61–68 amino acid residues), cysteine-rich, metal binding proteins that are widely distributed in a living organism.^1,2^ These proteins play a crucial role in the regulation of essential metal ions as Zn(II) and Cu(I/II), protection of cells from toxic metals such as Cd(II), Pb(II), Hg(II), Ag(I), etc., and oxidative damage.^1,3–5^ MTs are primary cytosolic proteins but they are also present in mitochondria and the nucleus, as well as existing extracellularly.^1,6–8^ They are expressed in various tissues, including the liver, kidney, small intestine, and brain, and their expression is regulated by various physiological and pathological conditions, such as heavy metal exposure, inflammation, and oxidative stress.^1,5^ There are four major isoforms of MTs (MT1–MT4), each with a distinct expression pattern and function. MT1, present in several subisoforms, and MT2 lack tissue specificity and are expressed in all kinds of cells at different levels and ratios, whereas MT3 and MT4 have been found mostly in the central nervous system and in stratified epithelial cells, respectively.^1,5,9,10^ For example, human MT has been found to be upregulated in several types of cancer, including liver, prostate, and breast cancer, suggesting a role for human MTs in the development and progression of these diseases.^11,12^

**Fig. 1:**
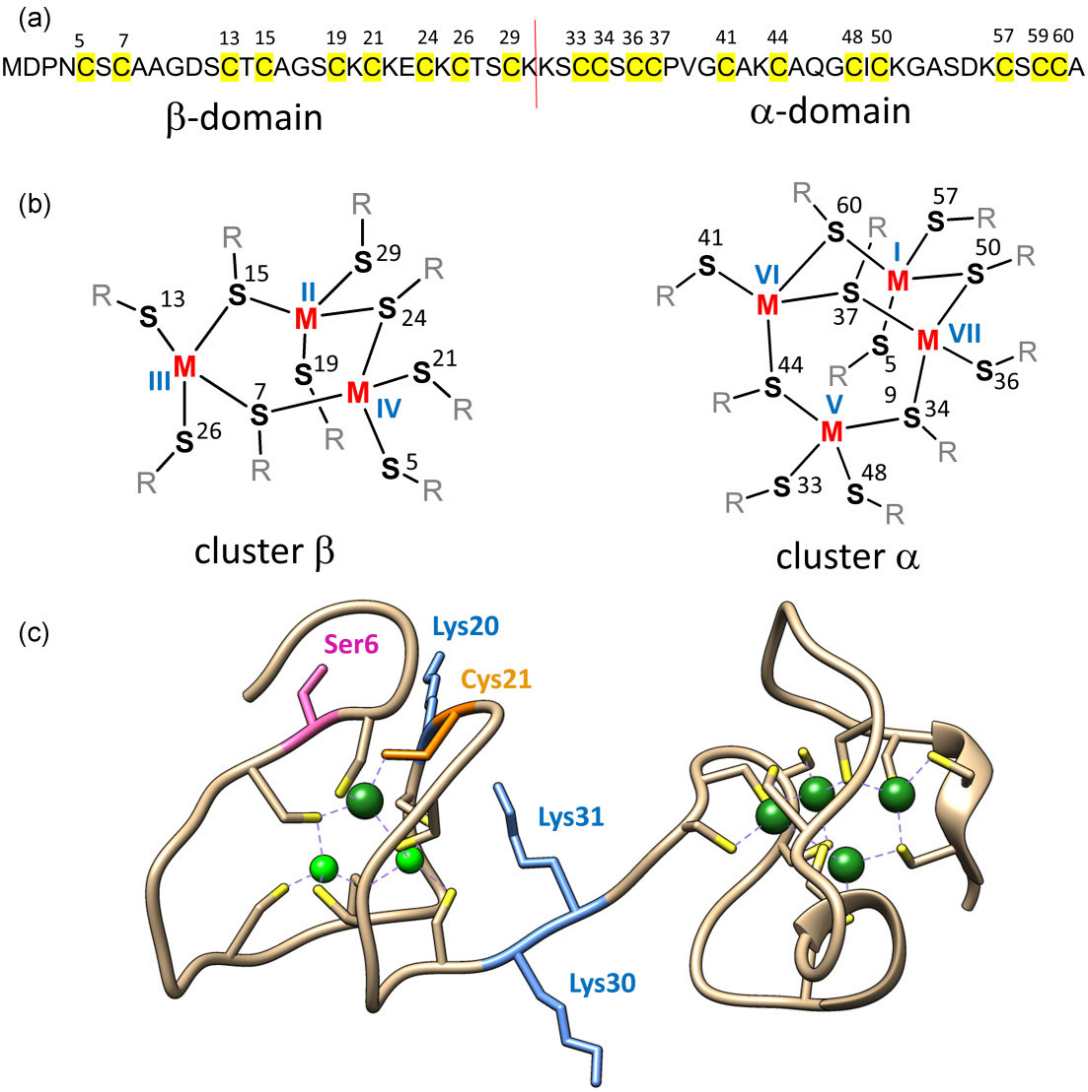
Sequence and structure of mammalian MT2. (a) Amino acid sequence of human MT2 used in this study. Yellow color and red line highlight cysteine residues responsible for metal binding and edge of α- and β-domains, respectively. (b) Structures of M_3_S_9_ (β) and M_4_S_11_ (α) clusters in rabbit MT2 determined by NMR (PDB: 1MRB, 2MRB),^14,15^ (c) The only crystal structure of mammalian MT (rat MT2, PDB: 4MT2), being a mixed Zn(II) and Cd(II) complex (Cd_5_Zn_2_MT2).^13^ Blue, orange and pink sticks represent lysine, cysteine, and serine residues mutated in this study, respectively. Green and light green spheres correspond to Cd(II) and Zn(II) ions, respectively, as present in PDB: 4MT2.

MTs contain two distinct domains, the N-terminal β-domain and the C-terminal α-domain (Fig. 1a). They contain 9 and 11 cysteine residues, which form two separate metal-thiolate clusters, M_3_Cys_9_ and M_4_Cys_11_, respectively [M refers to Zn(II) or Cd(II), Fig. 1b]. There is only one crystal structure of hepatic rat Cd_5_Zn_2_MT2 (PDB: 4MT2); however, several nuclear magnetic resonance (NMR) structures of isolated (separated) α- and β-domains from mammalian MT1–MT3 are available.^13–17^ It has been shown that isolated domains contain very similar structures, e.g. in X-ray studies, and physicochemical investigations have shown that they demonstrate comparable metal binding properties.^17–20^ MT domains have been used over the decades as convenient models, smaller than whole MTs, of studying metal folding and reactivity of MTs by numerous techniques including spectroscopies, electrochemistry, mass spectrometry (MS), and others.^1,21–29^ Indeed, careful analysis of X-ray structure shows, with one exception (Fig. 1c), lack of interdomain interactions, which has also been confirmed by NMR investigations.^30,31^ It should be underlined, however, that since the discovery of MTs, they have been characterized, almost exclusively, with Cd(II) due to the spectroscopic silence of Zn(II) ions despite their biological roles. Recent MS and molecular dynamics (MD) based deep studies on Zn(II) and Cd(II)-mediated MT2 folding have demonstrated that metalation mechanisms of these metal ions differ significantly, which calls into question many previous conclusions.^32,33^ Early studies demonstrated that seven Zn(II) ions bind to MTs with undistinguished high affinity with average *K*_d_ (*K*_d_^av^) of 10^−13^–10^−^^11^ M, while investigations with sensitive zinc fluorescent probes uncovered the differentiation of those affinities from low pico- to nanomolar range.^25,26,34–38^ This affinity differentiation results in coexistence of several Zn_4-7_MT species depending on molar Zn(II)-to-MT ratios, which have been shown to function as Zn(II) donors and acceptors controlling the structure and activity of numerous Zn(II)-dependent proteins.^39–44^ Proteomic approaches applied for whole MT2 indicated that a low affinity site is present in the β-domain, while initial pH-dependent studies on the synthetic β-domain indicated that its Zn(II) ions bind with low picomolar range (*K*_d_^av^ of 5 × 10^−^^12^ M at pH 7).^45,46^ This clearly shows that the connectivity of both domains may impact overall Zn(II) affinities of the binding sites and possibly the activity of isolated domains vs. whole protein. Indeed, the comparison of lysine and cysteine residues’ reactivity from both but separated domains (cumulative α+β effect) with the whole protein indicated such a difference.^45^ From this observations several important questions arise: (i) What are the molecular bases that change Zn(II) affinity and reactivity of isolated domains from their separated to linked state in the whole protein? (ii) Which internal effects present in a whole protein cause differentiation of particular Zn(II) ions’ thermodynamics? (iii) Where is the weakest binding site in the Zn_7_MT molecule located? And finally (iv), what are the biological consequences of zinc binding sites’ affinity differentiation?

Here, we experimentally test various possible—and identified in the crystal structure of rat Cd(II)/Zn(II)MT2—interactions within human MT2 and its domains to dissect the molecular basis of various affinity zinc sites. By combining mutagenesis, spectroscopic studies and steered molecular dynamics (SMD) simulations we show that the connection of α- and β-domains and subsequently formed intra- and interdomain interactions in MT2 differentiate Zn(II) ion affinities and determine its zinc buffering properties. We shed new light on MT folding mechanisms, energetics of Zn(II) (un)binding and structural features of zinc mammalian MTs, showing that any type of sequence or structure alteration affects Zn(II)-to-protein stability and impacts buffering properties.

## Results

### Purpose and molecular objects of the study

**Fig. 2:**
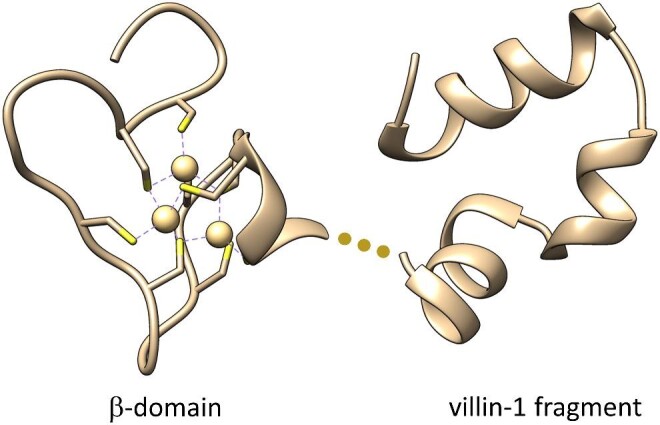
Schematic representation of hybrid protein βMT2-villin being a fusion of the β-domain of human metallothionein MT2 (PDB: 2MHU, Cd(II) complex) and the HP-35 subdomain of the headpiece of actin binding protein villin-1 (PDB: 1VII).^16,49^ Circles represent the KKS linker placed between fused elements (Supplementary Table S1).

The main aim of this study is to probe how alteration of some structural components of human zinc metallothionein-2 affect Zn(II)-to-protein affinity of their binding sites. Based on our and others’ previous observations, we initially focused on the impact of particular residues on intra- or interdomain interactions in MT2 on Zn(II) binding thermodynamics. Therefore in this study, besides the full MT2 sequence, we also investigated isolated domains (αMT2, βMT2) containing a short Lys-Lys-Ser (KKS) motif present between domains in a whole molecule (Supplementary Table S1). Secondly, to further elucidate the particular impact of Lys30 and Lys31 residues from that linker was examined by the generation of three Lys-to-Ala mutant proteins: K30A-MT2, K31A-MT2, and double K30A-K31A-MT2. We also prepared the Ser6-to-Ala mutant (S6A-MT2) because Ser6 is one of the few non-Cys residues conserved in all isoforms and we aimed to test its impact on Zn(II) binding properties in the β-domain (Fig. 1c). Our previous MS and MD-based data uncovered Cys21 as a key residue in completion of the β-cluster.^46^ Therefore, by its mutation (C21A-MT2), we wanted to analyse how the removal of this residue impacts the whole cluster formation. Because Lys20 is neighboring to Cys21 residues and overall lysines were shown to interact with cysteines in MTs and can modify the thiol pK_a_, we mutated that residue as well, obtaining the K20A-MT2 mutant (Fig. 1c, Supplementary Table S1).^1,13,47,48^ Finally, based on our previous results on the difference in Zn(II) metalation of the β-cluster in an isolated domain and whole protein, we constructed a fusion protein that contains the β-domain, a KKS linker and a small, compact protein with size comparable to the α-domain.^45^ For that purpose we chose the highly thermostable subdomain HP-35 (LSDEDFKAVFGMTRSAFANLPLWLQQHLLKEKGLF) of the chicken headpiece domain of villin-1 protein.^49^ HP-35 is the smallest naturally occurring polypeptide that folds autonomously in a globular structure with a well-packed hydrophobic core without any cofactor or disulfide bond.^50^ The putative structure of a such hybrid protein (βMT2-villin) is presented in Fig. 2 (Supplementary Table S1).

### Expression and purification of MT2-WT, MT2 α-domain, MT2 β-domain, and MT2-villin

Human MT2, its domains, their fusion and mutant proteins were expressed and purified using IMPACT intein mediated purification with the affinity chitin binding protein (CBP) system.^51^ This system was chosen because after intein DL-dithiothreitol (DTT)-assisted cleavage there are no remaining amino acid residues from the fusion protein. The expression and purification of the CBP fusions as well as profiles of purified MTs were analysed using SDS-PAGE. MT visualization was achieved by labeling with biarsenical probes and representative SDS-PAGE (sodium dodecyl sulfate-polyacrylamide gel electrophoresis) gel is presented in Supplementary Fig. S1.^52,53^ Because a fluorescence signal was developed only for MT and its fusions, we were able to quickly ascertain the efficiency of fusion production as well as all purification steps. To ensure metal homogeneity the MTs released from CBP fusion were demetalated to apo-MTs by acidification until pH ∼2.2 with 7% HCl and concentrated using a 3 kDa Amicon centrifugal filter. The concentrated apo-proteins were purified on a size exclusion chromatography (SEC) column equilibrated with 10 mM HCl to avoid protein oxidation during separation. The identity of all obtained proteins was checked by MS and molecular masses are provided in Supplementary Table S2. It should be noted that for some experiments (discussion in the following text) metal-free proteins were used directly and in these cases only freshly prepared proteins were used. It is highly important not to use apo-forms of MTs that have been stored without bound metal ions because they easily oxidize.^54^

In order to prepare holo-forms, the fractions containing freshly prepared apo-proteins were collected and metalated by adding excess of ZnSO_4_ (up to 115% of expected loading state) in the presence of 1 mM Tris(2-carboxyethyl)phosphine (TCEP) under a nitrogen blanket. For metalation the pH was increased to 8.6 using 1 M Tris base. Zn(II)-loaded samples were concentrated using a 3 kDa Amicon centrifugal filter and holo-proteins were purified on a SEC column equilibrated with 20 mM Tris pH 8.6. The SEC fractions having holo-MTs were concentrated and aliquots were stored at −80°C until further use. Of note, TCEP used during the metalation process is a very weakly Zn(II) binding reducing agent in contrast to DTT and can be safely used because it does not compete for Zn(II) under the used conditions and it is removed in subsequent purification on the SEC column.^55,56^ Apart from DTNB-based [5,5ʹ-dithiobis(2-nitrobenzoic acid)] measurements, TCEP was also added in the experiments to protect thiol(ates).^55,57^

### Zn(II) binding stoichiometries of investigated zinc proteins

The stoichiometry of Zn(II) complexes obtained after reconstitution and purification of holo-forms was determined using two different approaches. In the first one, two spectroscopic assays were applied for total thiol(ates) and Zn(II) concentration determination. Thiol(ates) were determined by DTNB in the presence of ethylenediaminetetraacetic acid (EDTA) and Zn(II) by PAR [4-(2-pyridylazo)resorcinol] in the presence of DTNB.^57,58^ Thiol(ate) concentration was then transformed to protein concentration. It was possible to assume that all Cys residues were reduced due to fresh reduction and preparation. The determined molar ratios between Zn(II) and protein concentrations are presented in Table 1. In the second approach, protein samples were wet mineralized in nitric acid and analysed for zinc content by inductively coupled plasma (ICP) MS. Obtained Zn(II) molar concentrations were divided by molar protein concentrations to obtain ratios analogous to spectroscopic assays (Table 1). Results from both approaches are highly convergent despite methodological differences. Data obtained for α- and β-domains indicate their full, and expected saturation of 4 and 3 Zn(II) mol. eq., respectively. MT2 (WT-wild type), S6A-MT2, K20A-MT2, K30A-MT2, K30A-MT2, and K30_K31A-MT2 bind 7 Zn(II) mol. eq. as indicated by average Zn(II)/protein values (Table 1). Only in the case of C21A-MT2 obtained values vary significantly between preparations, which was not observed for other zinc proteins, where the final value, 5.5 ± 0.5 (Zn(II)/protein), was averaged from three independent isolations. It is far from the expected value of 7 Zn(II) mol. eq. as in the case of other single and double mutants of MT2. Interestingly, stoichiometric analysis of C21A-MT2 saturated independently with Cd(II) and purified later under the same conditions as Zn(II) complex gave 6.1 ± 0.1 Cd(II) mol. eq. per protein (value determined with PAR).^58^ Finally, the βMT2-villin hybrid indicated the presence of 3 Zn(II) mol. eq. as expected for protein containing only the βMT domain (Table 1).

**Table 1.** ICP and spectroscopic analysis of Zn(II) (or Cd(II) when indicated)^a^ content in reconstituted MT2, mutants and isolated domains obtained in this study.

Protein	Zn(II)/protein ICP	Zn(II)/protein spectroscopic assays	Zn(II)/protein average
MT2 (WT)	7.1 ± 0.1	7.0 ± 0.2	7.0 ± 0.3
αMT2 (α-domain)	3.9 ± 0.2	4.1 ± 0.2	4.0 ± 0.3
βMT2 (β-domain)	2.6 ± 0.3	3.2 ± 0.1	2.9 ± 0.4
βMT2-villin	2.9 ± 0.1	3.0 ± 0.2	2.9 ± 0.3
S6A-MT2	6.9 ± 0.1	7.1 ± 0.1	7.0 ± 0.2
K20A-MT2	6.8 ± 0.1	7.1 ± 0.1	6.9 ± 0.2
C21A-MT2	5.3 ± 0.3	5.7 ± 0.2	5.5 ± 0.5
	6.2 ± 0.2^a^	6.1 ± 0.2^a^	6.1 ± 0.2^a^
K30A-MT2	7.1 ± 0.2	6.8 ± 0.1	6.9 ± 0.3
K31A-MT2	6.8 ± 0.1	7.2 ± 0.2	7.0 ± 0.3
K30A_K31A-MT2	6.8 ± 0.2	7.1 ± 0.1	6.9 ± 0.3

^a^Values correspond to Cd(II) analysis.

**Fig. 3:**
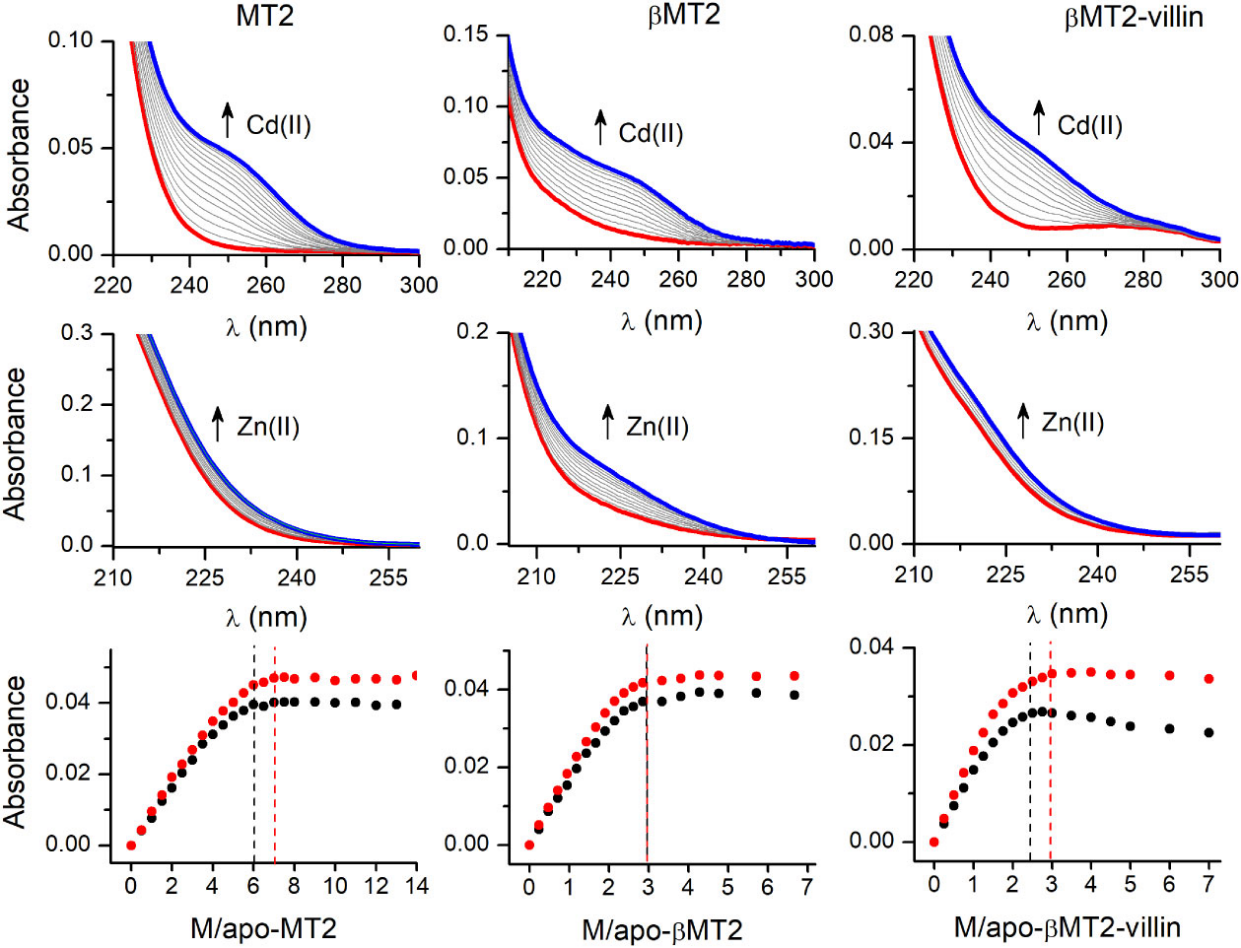
UV-visual titrations of Cd(II) (top row) and Zn(II) (middle row) to metal-free MT2 (1 µM), βMT2, and βMT2-villin (2 µM) in 50 mM borate pH 7.4, 100 mM NaClO_4_, 100 µM TCEP. Red and blue lines correspond to spectra of metal-free and fully saturated protein, respectively. Spectra above saturation were omitted for clarity. The bottom row indicates absorbances at 220 and 240 nm for Zn(II) (black circles) and Cd(II) (red circles) titrations. Black and red vertical lines indicate metal-to-protein ratios where absorbance reaches saturation for Zn(II) and Cd(II), respectively.

Electronic spectroscopy in the far ultraviolet (UV) range is the most common technique used to study the metalation mechanisms and metal state of MTs.^22,24^ MT spectra demonstrate characteristic ligand-to-metal charge transfer (LMCT) bands in that region. Therefore, LMCT bands formed during Zn(II) and Cd(II) step titrations to metal-free MT2, βMT2 domains, and βMT2-villin in the range of 200–300 nm were used for metalation status monitoring. Firstly, 50 mM borate buffer (100 mM NaClO_4_, pH 7.4) with 100 µM TCEP was blanked followed by the addition of metal-free protein (1 µM MT2 or 2 µM βMT2/βMT2-villin) and then it was step-titrated with ZnSO_4_ or CdSO_4_ mole equivalents in amounts twice as high as signal saturation. MT2, βMT2 domain, and βMT2-villin were titrated with Zn(II) and Cd(II) to compare the metalation processes. The importance of that experiment is outlined in the discussion. The top row of Fig. 3 demonstrates spectra in the UV range of proteins titrated with CdSO_4_. The obtained spectra are highly similar to those observed previously for MT2 and its β-domain. Worth indicating is a significantly wider range of LMCT bands for the β-domain than for WT. Interestingly, spectra for βMT2-villin, which contains the β-domain, are more similar to the WT protein than the isolated domain. The middle row of Fig. 3 shows analogous titrations with ZnSO_4_ indicating analogous similarities as in the case of Cd(II) titration. βMT2-villin titrations with Zn(II) remain similar to that of WT, while the isolated β-domain shows substantial differences. The comparison of absorbance increases for Cd(II) and Zn(II) titrations shown in the bottom row of Fig. 3 indicates some differences between proteins. WT protein, when titrated with Cd(II), indicates saturation at 7 Cd(II) mol. eq. (red line) when monitored at 240 nm, while for Zn(II) titration, absorbance reaches saturation ∼6 mol. eq. (black line). The same behavior was observed in previous studies on WT.^33^ In the case of the β-domain, which binds three metal ions, saturation for Zn(II) and Cd(II) occurs at a similar value of 3 mol. eq. Saturation of βMT2-villin occurs between the two previous proteins. Cd(II) saturates absorbance at 3 mol. eq., while Zn(II) does so at ∼2.5 mol. eq., indicating significant similarity to WT with the difference that the saturation occurs with a smaller amount of metal equivalents.

### Competition of MT2 and its mutants with PAR

**Fig. 4:**
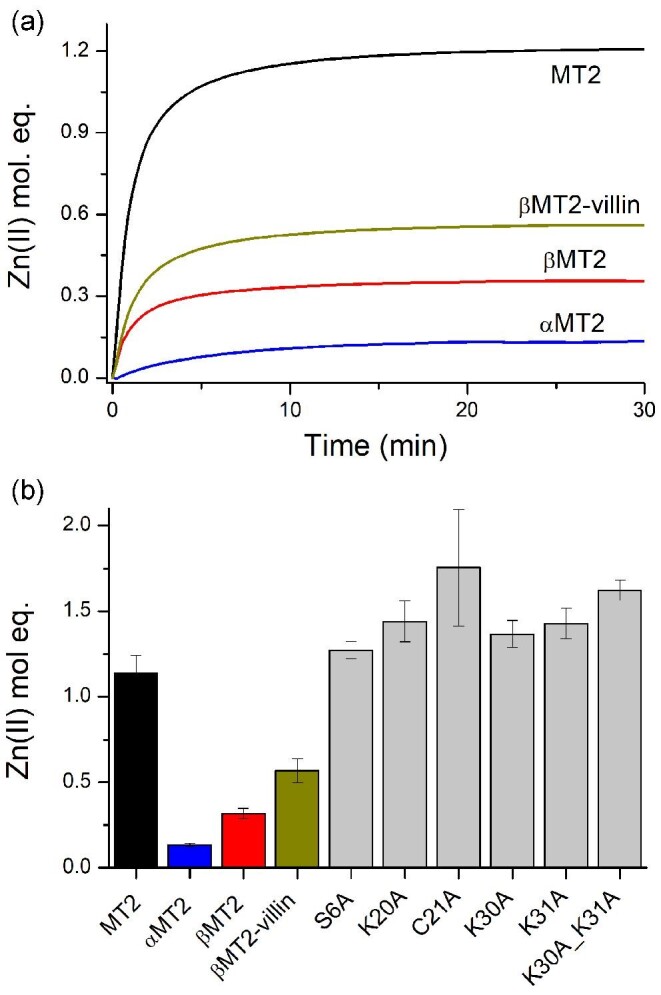
Competition of MT2, its domains, and mutants (1.7 µM) with 200 µM PAR in 50 mM Na^++^HEPES buffer pH 7.4 (0.1 M NaCl) in the presence of 200 µM TCEP.^55^ (a) Exemplary kinetics of Zn(II) transfer to PAR monitored at 492 nm and then recalculated to Zn(II) mole equivalents using molar absorption coefficient 71 500 M^−1^ cm^−1^.^58^ (b) The comparison of Zn(II) mole equivalents transferred to PAR among all studied proteins under used conditions.

Investigation of MT proteins competition with chromogenic and fluorescent chelating agents for the determination of stability (binding or dissociation) constants became common due to these approaches’ simplicity and high convenience.^1,34,35,37,38,59^ For instance, PAR is one of the popular chromogenic probes applied for studying Zn(II) (un)binding to MTs and other zinc proteins.^41,59–62^ In this study, it was used to determine dissociation constants of Zn(II) ions bound to MT2 or its mutants, fusions, and domains; however, it should be noted that PAR is unable to remove all seven Zn(II) ions, and competes only for the weakest binding site(s). Quantitative PAR use for zinc protein applications was described by Kocyła *et al*. and it was used here with some modifications.^58^ Here, micromolar concentrations of purified fully Zn(II)-loaded protein were incubated with 200 µM PAR for sufficient equilibration time (∼30 min) in the presence of TCEP to protect thiolates (and thiols formed upon Zn(II) transfer) against air oxidation, and the increase of Zn(PAR)_2_ complex formation was measured as the absorbance increase at 492 nm.^62^ Figure 4a shows kinetics of Zn(II) transfer between α- and β-domains and full MT2 to PAR excess as reaction examples. These data indicate major differences in the percentage of Zn(II) transferred between the full protein and its domains. Transfer from the β-domain was more efficient than for the α-domain, indicating lower Zn(II) affinity to the former. Interestingly, numbers of Zn(II) equivalents transferred from βMT2-villin are comparable to that of the β-domain, indicating similar Zn(II) affinities of the weakest zinc site in both molecules (Fig. 4b). All MT2 single and double mutants investigated here indicated either similar to WT or higher Zn(II) transfer to PAR. Among them, the most comparable to WT is S6A-MT2 and then K30A-MT2, while the most significant difference was observed for the C21A-MT2 mutant and then K30_K31-MT2.

In order to calculate the first dissociation constant (*K*_d1_) values, at the first point, absorbances at 492 nm were converted to Zn(PAR)_2_ complex concentrations using the molar absorption coefficient 71 500 M^−1^ cm^−1^ (Equation (1)).^58^ Then, based on that value, total PAR and MT2 concentrations (βMT2 was chosen here as an example), free PAR, Zn_3_βMT2, and Zn_2_βMT2 concentrations being in equilibrium were calculated (see Supplementary data for calculation details). It should be noted that this binding model was applied to those proteins for which up to 1 Zn(II) mol. eq. was transferred (Fig. 4b).


(1) \begin{eqnarray*}
{\mathrm{Z}}{{\mathrm{n}}}_3{\mathrm{\beta MT}}2 + 2{\mathrm{PAR}} \rightleftharpoons {\mathrm{Z}}{{\mathrm{n}}}_2{\mathrm{\beta M}}{{\mathrm{T}}}2 + {\mathrm{Zn}}{\left( {{\mathrm{PAR}}} \right)}_2
\end{eqnarray*}

Knowledge on reactants’ concentrations from Equation (1) allowed calculation of the exchange constant *K*_ex1_ (Equation (2)). This value allows one to calculate *K*_d1_ of MT protein based on previously published by our group dissociation constant of Zn(PAR)_2_ complex, *K*_d12_^PAR^ = 7.1 × 10^−13^ M^2^ following Equation (3).


(2) \begin{eqnarray*}
K_{{\mathrm{ex}}1} = \frac{{\left[ {{\mathrm{Zn}}{{\left( {{\mathrm{PAR}}} \right)}}_2\left] \cdot \right[{\mathrm{Z}}{{\mathrm{n}}}_2\beta {\mathrm{MT}}2} \right]}}{{\left[ {{\mathrm{Z}}{{\mathrm{n}}}_3\beta {\mathrm{MT}}2} \right] \cdot {{\left[ {{\mathrm{PAR}}} \right]}}^{2}}}
\end{eqnarray*}

(3) \begin{eqnarray*}
{K}_{{\mathrm{d}}1} = \frac{{\left[ {{\mathrm{Z}}{{\mathrm{n}}}_2{\mathrm{\beta MT}}2} \right]{{\left[ {{\mathrm{Zn}}\!\left( {{\mathrm{II}}} \right)} \right]}}_{{\mathrm{free}}}}}{{\left[ {{\mathrm{Z}}{{\mathrm{n}}}_3{\mathrm{\beta MT}}2} \right]}} = {K}_{{\mathrm{ex}}1} \times {K}_{{\mathrm{d}}12}^{{\mathrm{PAR}}}
\end{eqnarray*}

As pointed out earlier, this approach can be used only for those proteins which demonstrate transfer up to 1 Zn(II) mol. eq. For the remaining ones (Fig. 4b), for which Zn(II) transfer occurs between 1 up to 2 Zn(II) mol. eq. different binding model must be considered. It includes transferring two of the weakest Zn(II) ions from the protein. In the case of MT2, Zn_7_MT2 and Zn_5_MT2 are taken as a substrate and product, respectively (Equation (4)). It does not mean that the Zn_6_MT2 species is not formed as an intermediate, but it is impossible to determine its concentration with this data set, therefore it was omitted in this approach.


(4) \begin{eqnarray*}
{\mathrm{Z}}{{\mathrm{n}}}_7{\mathrm{MT}}2 + 4{\mathrm{PAR}} \rightleftharpoons {\mathrm{Z}}{{\mathrm{n}}}_5{\mathrm{MT}}2 + 2{\mathrm{Zn}}{\left( {{\mathrm{PAR}}} \right)}_2
\end{eqnarray*}

The constant, which describes Zn(II) transfer process, is an exchange constant *K*_ex12_, defined by Equation (5). This value allows calculating *K*_d12_ of MT protein based on previously *K*_d12_^PAR^ = 7.1 × 10^−13^ M^2^ following Equation (6). Calculation details are presented in the Supplementary data.


(5) \begin{eqnarray*}
K_{{\mathrm{ex}}12} = \frac{{\left[ {{\mathrm{Z}}{{\mathrm{n}}}_5{\mathrm{MT}}2} \right]{{\left[ {{\mathrm{Zn}}{{\left( {{\mathrm{PAR}}} \right)}}_2} \right]}}^2}}{{\left[ {{\mathrm{Z}}{{\mathrm{n}}}_7{\mathrm{MT}}2} \right] \cdot {{\left[ {{\mathrm{PAR}}} \right]}}^4}}
\end{eqnarray*}

(6) \begin{eqnarray*}{K}_{{\mathrm{d}}12} = \frac{{\left[ {{\mathrm{Z}}{{\mathrm{n}}}_5{\mathrm{MT}}2} \right]\left[ {{\mathrm{Zn}}\!\left( {{\mathrm{II}}} \right)} \right]_{{\mathrm{free}}}^2}}{{\left[ {{\mathrm{Z}}{{\mathrm{n}}}_7{\mathrm{MT}}2} \right]}} = {K}_{{\mathrm{ex}}12} \times {\left( {{K}_{{\mathrm{d}}12}^{{\mathrm{PAR}}}} \right)}^2\end{eqnarray*}

Because *K*_d12_ is a cumulative constant of two events (*K*_d12_ = *K*_d1_⋅*K*_d2_), its a direct comparison to *K*_d1_ values for proteins, which demonstrate less than 1 Zn(II) mol. eq. transfer is impossible. Therefore, we used here an average constant of these two processes, *K*_d12_^av^, which still cannot be directly compared to *K*_d12_ but at least has the same order of magnitude and unit (Table 2). It is obtained by the square root of *K*_d12_ according to Equation (7).


(7) \begin{eqnarray*}
K_{d12}^{\ av} = \sqrt {{K}_{d12}} \
\end{eqnarray*}

**Table 2.** Dissociation constants of Zn(II) complexes with MT2 domains or proteins determined spectrophotometrically or spectrofluorometrically in competition with PAR or ZnAF-2F at pH 7.4 (NaCl = 0.1 M) in the presence of 200 µM TCEP^a^.

	−log*K*_d1_	−log*K*_d12_^av^	−log*K*_d1_
Protein	(PAR)	(PAR)	(ZnAF-2F)
MT2		10.40 ± 0.05	8.40 ± 0.03
αMT2	12.19 ± 0.02		>9.3
βMT2	11.36 ± 0.03		>9.7
βMT2-villin	10.64 ± 0.05		8.55 ± 0.06
S6A-MT2		10.30 ± 0.03	Not determined (n.d.)
K20A-MT2		10.16 ± 0.07	n.d.
C21A-MT2		9.8 ± 0.2	n.d.
K30A-MT2		10.22 ± 0.07	n.d.
K31A-MT2		10.17 ± 0.05	n.d.
K30A_K31A-MT2		10.00 ± 0.04	n.d.

^a^In the case of PAR, depending on Zn(II) mole equivalents mobilized during competition *K*_d1_ [up to 1 Zn(II) mol. eq.] or *K*_d12_^av^ [between 1 and 2 Zn(II) mol. eq.] values were calculated.

All calculated *K*_d1_ or *K*_d12_^av^ values are presented in Table 2. The lowest dissociation constants are for the α- and then for the β-domain. It indicates that domain isolation impacts thermodynamics of the system (see discussion in the following text). Although βMT2 and βMT2-villin bound the same number of Zn(II) ions, their *K*_d1_ differs by ∼0.7 orders of magnitude. Regarding mutants, all of them demonstrate various but lower Zn(II) affinity (higher *K*_d_ values) compared to MT2 (WT). The most similar to WT is S6A-MT2 mutant. Among all lysine mutants, K20A-MT2 demonstrates the tightest zinc affinity, while the weakest is for K30A_K31A-MT2. However, the MT2 mutant, which binds Zn(II) the most loosely among all studied proteins, is C21A-MT2 (Table 2). Determination of *K*_d12_^av^ in this case is burdened with the largest error due to various amounts of Zn(II) found in isolations (the constant was determined using various isolations).

**Fig. 5:**
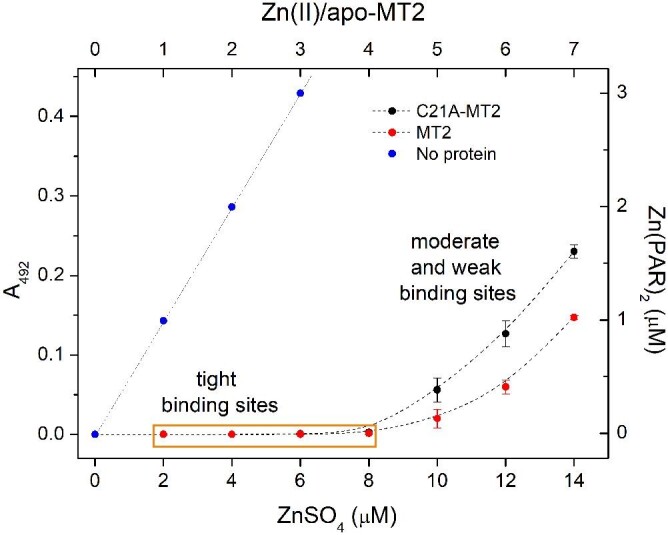
Relationship between absorbance increase of PAR at 492 nm upon ZnSO_4_ addition to metal-free WT-MT2 (red circles) or C21A-MT2 (black circles). 2 µM metal-free protein was mixed with 0–14 µM of ZnSO_4_ (0–7 Zn(II) mol. eq. counted per MT2) and then subjected to 200 µM PAR for competition in the presence of 200 µM TCEP. Blue dots represent the absorbance increase for the PAR sample titrated with ZnSO_4_ in the absence of protein. 50 mM Na^++^HEPES buffer pH 7.4 with 0.1 M NaCl was used as a buffer.

Because C21A-MT2 is the most different mutant from all those investigated, we aimed to test how particular Zn(II) ions bound to the protein differ from those of WT. For that purpose metal-free forms of both proteins were partially or fully saturated with ZnSO_4_ and then incubated with 200 µM PAR in order to analyse how much of Zn(II) bound to the protein is capable of exchange (Fig. 5). This experiment shows that in both cases 4 Zn(II) mol. eq. are bound tightly to the proteins with undistinguished affinities under the used conditions. It means that PAR is unable to compete with the four most tightly bound Zn(II) ions. Competition with PAR after addition of 5 Zn(II) mol. eq. indicates that more metal ions are transferred from C21A-MT2 than from WT. This difference is even more visible for 6 Zn(II) mol. eq., and this trend is continued for 7 Zn(II) mol. eq., as shown in Fig. 5. At this ratio, MT2 mobilizes 1.03 Zn(II) mol. eq., which is almost the same value as in the case of reconstituted zinc protein treated with PAR excess (Fig. 4a).

### Competition of MT2 and its mutants with ZnAF-2F

Although PAR is commonly used as a probe for studying Zn(II) dissociation or association, its major drawback is the formation of ZnL_2_ complexes, which complicates calculations or increase the chance for formation of intermediates affecting calculations (see the following text).^58^ Therefore, other chromogenic or fluorescent probes that form a 1:1 complex with Zn(II) may serve as an alternative. In 2007, we applied a fluorescent probe, FluoZin-3, for determination of stability constants of the Zn(II)-MT2 system.^38^ It binds Zn(II) with 1:1 stoichiometry, nanomolar affinity, and demonstrates high sensitivity toward Zn(II). Here, we applied the ZnAF-2F fluorescent probe, which is comparable to FluoZin-3 regarding its Zn(II) binding and optical properties.^63,64^

**Fig. 6:**
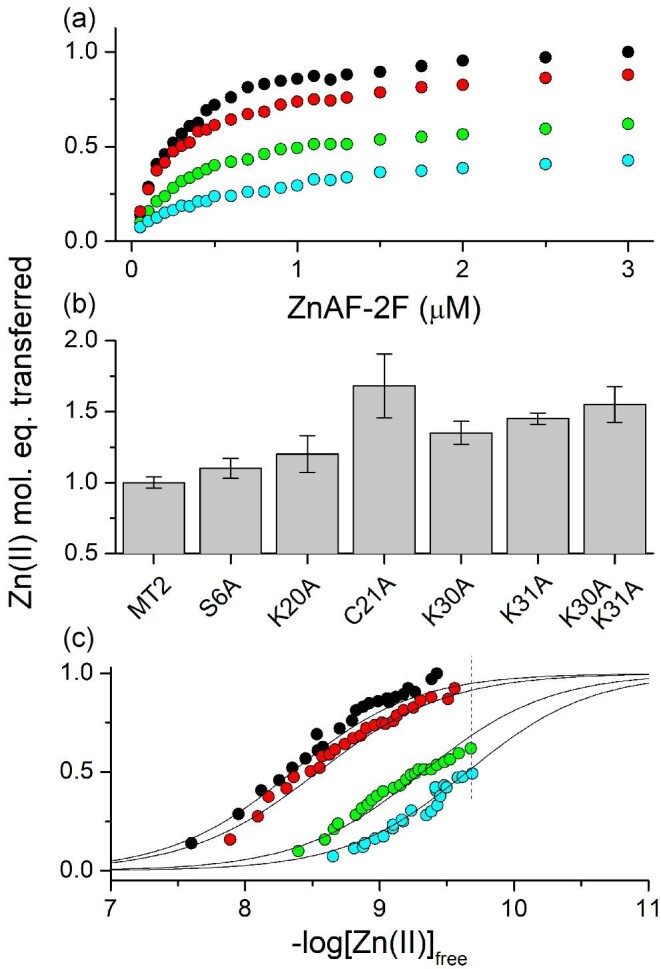
The competition between MT2, its mutants and isolated domains (0.5 µM) with 0–3 µM ZnAF-2F in 50 mM Na^++^HEPES pH 7.4, 100 mM NaCl, 500 µM TCEP. (a) Zn(II) mole equivalents transferred from MT2 (black), βMT-villin (red), βMT2 (green), and αMT2 (blue) to various ZnAF-2F concentrations over 1.5 h. (b) Comparison of MT2 with its single or double mutants regarding their competition with 3 µM ZnAF-2F (continues from upper graph). (c) Comparison of fractions from top panel presented as a function of free Zn(II) concentration fitted to Hill's equation. Dashed line indicates −log[Zn(II)]_free_ limits for ZnAF-2F under used conditions. Colors correspond to top graph.

The use of ZnAF-2F has important advantages compared to PAR—not only more favorable Zn(II) complex stoichiometry, but also probe sensitivity and slightly higher, compared to PAR, affinity. The latter two properties allows its concentration to be minimized from hundreds to a few micromoles and MT concentration to be decreased as well. Lower probe excess over MT allows better control of the competition reaction, reduces calculation errors and lowers the chance for nonspecific or specific interactions with protein (see the following text). Here, we used Zn(II)-MT2 at 0.5 µM, while ZnAF-2F varied from 0 to 3 µM. In the first experiment Zn(II)-loaded MT2, its domains and mutants were incubated with increasing concentration of ZnAF-2F for 2 h. Figure 6a demonstrates how the number of Zn(II) transferred equivalents depends on the probe concentrations for MT2, α- and β-domains, and βMT2-villin as examples. It shows that the highest transfer is observed for MT2 and βMT-villin and reaches values of 1 and 0.88 in the presence of 3 µM ZnAF-2F. For the α- and β-domains this value was 0.43 and 0.62, respectively. Figure 6b compares the transferred Zn(II) mole equivalents for WT with the rest of 1- or 2-point mutants, which without exception donate more Zn(II), indicating their lower affinity. The largest transfer, 1.68 Zn(II) mol. eq. at 3 µM ZnAF-2F, was observed for C21A, although with a significant error. The next weakest was the double mutant K30A_K31A-MT2, while S6A-MT2 was the most similar to WT. It should be noted that the ZnAF-2F fluorescence increase during incubation with zinc proteins was calibrated to Zn(II) mole equivalents using independent total protein oxidation with dithiodipyridine and total Zn(II) release.

Determination of any stability constant with a zinc fluorescent probe requires its internal calibration. For that purpose samples from the earlier-described experiments (only those with lower transfer than WT) were first saturated with excess ZnSO_4_ (*F*_max_) and finally minimal fluorescence (*F*_min_) was measured after the addition of EDTA excess, whose affinity toward Zn(II) is more than four orders of magnitude higher than ZnAF-2F. To obtain free Zn(II) concentrations {[Zn(II)]_free_} all fluorescence values were used with known *K*_d_^ZnAF-2F^ for Zn-ZnAF-2F according to Equation (8).^38^


(8) \begin{eqnarray*}
{\left[ {{\mathrm{Zn}}\!\left( {{\mathrm{II}}} \right)} \right]}_{{\mathrm{free}}} = K_{\mathrm{d}}^{{\mathrm{ZnAF}} - 2{\mathrm{F}}}\ \cdot \frac{{\left[ {{{\mathrm{F}}}_{{\mathrm{max}}} - {\mathrm{F}}} \right]}}{{\left[ {{\mathrm{F}} - {{\mathrm{F}}}_{{\mathrm{min}}}} \right]}}
\end{eqnarray*}

Calculated [Zn(II)]_free_ values allow the Zn(II) transfer described in Fig. 6a and b to be presented in a new way (Fig. 6c), which also allows one to calculate *K*_d1_ values of investigated proteins. The *K*_d1_ values were actually obtained by Zn(II) transfer to Hill's equation (Table 2). The data indicate that among studied proteins the lowest −log*K*_d1_ of the weakest zinc site is present in MT2 (8.4), which corresponds to the previous results obtained with FluoZin-338; slightly tighter is the βMT2-villin with −log*K*_d1_ = 8.7. Isolated domains demonstrate significantly higher affinities and exact values cannot be determined under the applied conditions. However, Hill's equation fitted to obtained data, assuming a similar Zn(II) transfer pattern as in the case of the previous two proteins, allows one to estimate −log*K*_d1_ values, which are 9.3 and 9.7 for the β- and α-domain, respectively. It should be noted that these two values might be underestimated and actual −log*K*_d1_ values can be even higher, although with the same affinity order. Interestingly, all Hill's coefficient values obtained in data fitting are similar to each other and vary around 1.0. A coefficient value of 1.1, above the average, was found for MT2.

### Reactivity of MT proteins with DTNB

The competition with chromogenic agents is the best approach for the investigation of metal-to-MT affinities, especially in the case of complexes with spectroscopically silent Zn(II). However, besides that method, there are several others to probe the presence of weak or reactive metal binding site(s) in MTs. One such approach is the oxidation of thiolates or thiols (if they are not bound to metal or spontaneously deprotonated) in the case of metal complexes by DTNB at equimolar to protein concentrations. In this case, the velocity of DTNB reduction observed by absorbance increase of the product (TNB^−^) at 412 nm according to the first-order kinetic rate represents natural or enhanced (by truncation) zinc sites’ (ZnCys_4_) tendencies for oxidation. As shown for human MT2, this tendency is inversely proportional to the affinity of metal in its binding site.^38,45,65^ Moreover, this assay is also able to monitor Zn(II) transfer from MT to buffer components or Zn(II) acceptors. It is because Zn(II)-depleted sites are more prone to oxidation due to the presence of free thiols or weak zinc sites.^38,66^

**Fig. 7:**
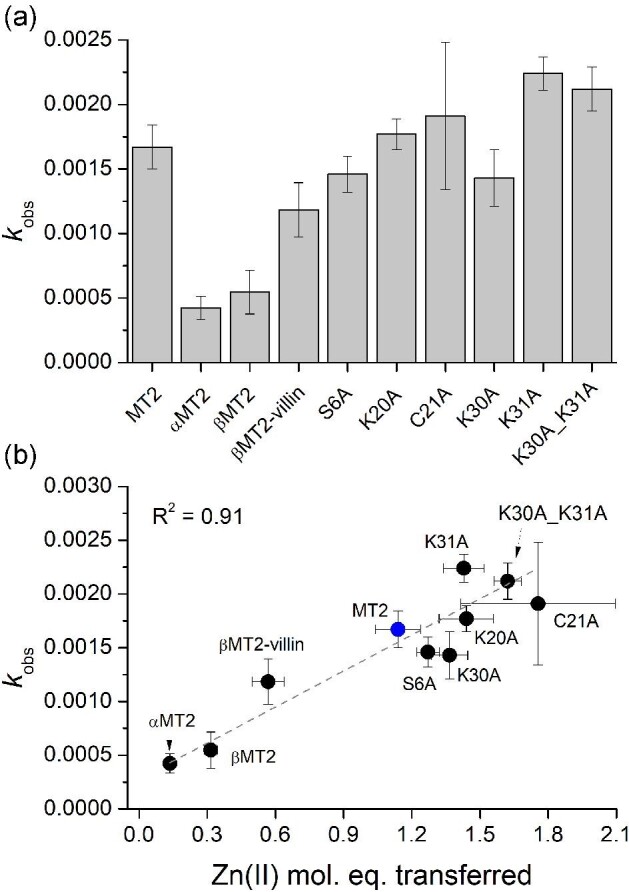
Oxidation of MT2, its domains and mutants (1.7 µM) with 2 µM DTNB in 50 mM Na^++^HEPES buffer, pH 7.4, 0.1 M NaCl. (a) Comparison of *k*_obs_ values for all investigated zinc proteins. (b) Comparison of obtained *k*_obs_ values with Zn(II) mole equivalents transferred to PAR from Fig. 4 (Table 2).

In this study MT2 and its mutants or domains were used at 1.7 µM concentrations, while DTNB was applied in a very slight excess of 2 µM. Depending on the protein used, the kinetic reaction was carried out from 50 to 120 min. Supplementary Fig. S2 presents examples of DTNB reduction kinetics. Under the applied conditions for all cases, besides the α-domain, kinetics were monophasic and the absorbance increase fitted the first-order kinetic rate equation to determine *k*_obs_ values.^38^ Figure 7a presents the comparison of obtained *k*_obs_ values for all studied proteins and domains. The slowest rate of DTNB reaction was observed for the α- then the β-domain and βMT2-villin, while the fastest rate was for K31A-MT2 and then K30AK31A-MT2. In the case of C21A-MT2 the oxidation rate varied significantly from one protein to another, meaning that purification products were more or less prone to DTNB oxidation. Therefore, for that mutant, *k*_obs_ has significantly higher error compared to the other proteins. It is even more visible when *k*_obs_ is compared with the number of Zn(II) equivalents transferred to the PAR (Fig. 7b) during equilibration (see earlier). This linear correlation (*R*^2^ = 0.91) shows the trend between thiolate reactivity (oxidation tendency) and the number of Zn(II) ions transferred from the protein for all investigated zinc proteins.

### SMD to investigate the unbinding pathways of Zn(II) in Zn_7_MT2

Previous studies using MS and MD simulations successfully identified the weakest Zn(II) site in the β-domain of Zn_7_MT2, although its location varied depending on the methodology used.^33,46^ In order to clarify these unresolved issues, we examined how Zn(II) unbinds from the β-domain in the full length Zn_7_MT2 and determined the energetic contributions of individual Zn−S bonds to each metal site. To this end, 10 independent SMD simulations were conducted for each Zn(II) site.

**Fig. 8:**
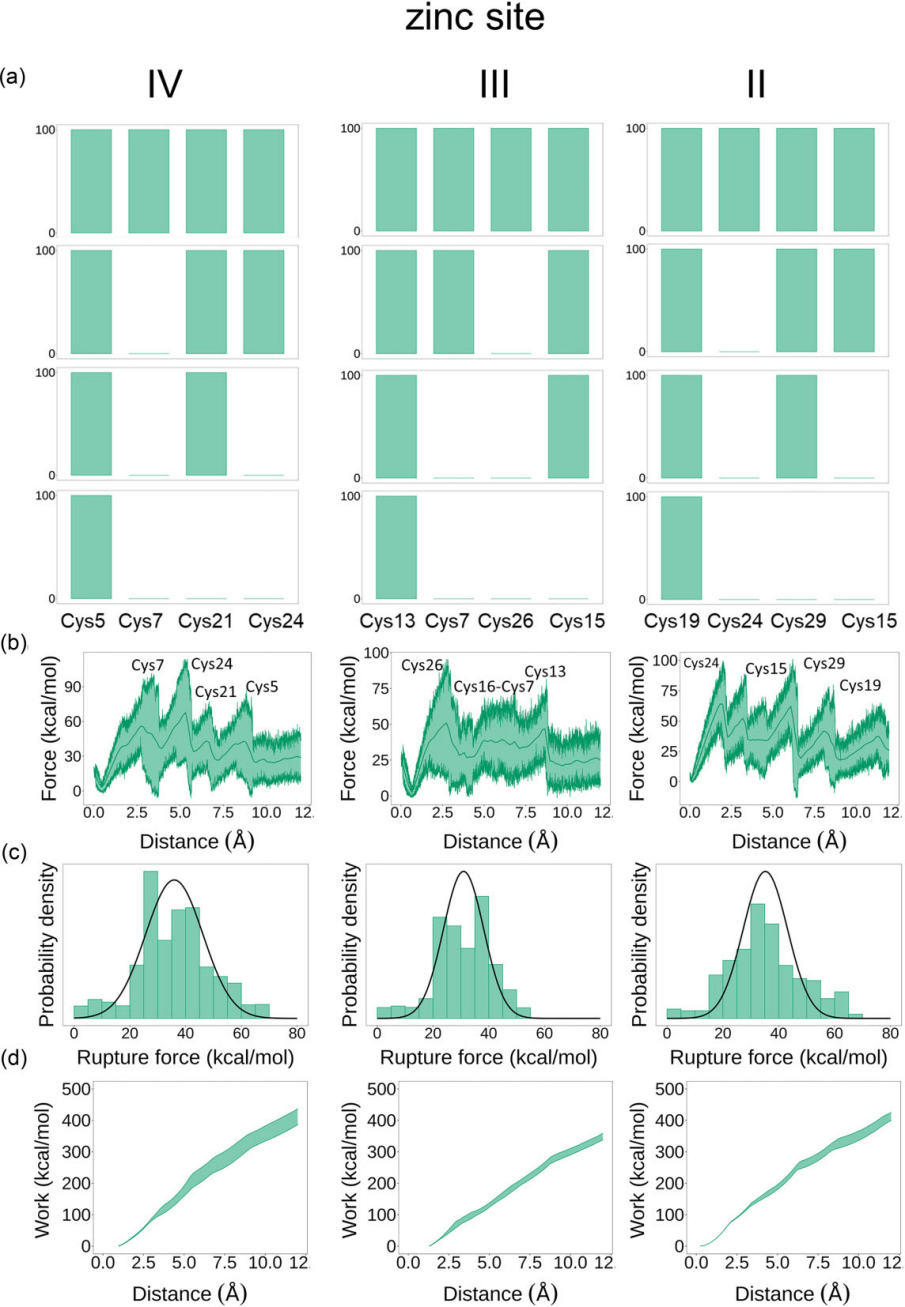
Steered MD simulations for the Zn_7_MT2 system. (a) Stepwise Zn−S bond dissociation for each zinc site in the β-domain. Formation and disruption of the Zn−S bond are represented by the presence and absence of a bar, respectively. (b) Force-extension curves derived from the SMD simulations. (c) Rupture force histograms for each zinc site. (d) Work profiles as a function of the Zn−S distance.

SMD are a type of MD simulations that involve applying an external force to a molecule to study its response to an external force. In this work, SMD simulations were used to induce Zn(II) dissociation from Zn_7_MT2. We identified two different unbinding pathways for each zinc site, that is sites IV, II, and III (Fig. 1b, Supplementary Table S3). As Zn(II) dissociates, Zn−S bonds are broken, and thus we can monitor the strength of each of these bonds and elucidate a microscopic unbinding pathway according to Equations (9) and (10). In Fig. 8a, the Zn−S dissociation pathways for zinc sites IV, II and III are shown, revealing which Zn−S bonds are being broken as Zn(II) dissociates and elucidating the microscopic unbinding pathways. Initially, each Zn(II) ion was bound by four Cys residues, and as the simulation proceeds Zn(II) dissociates step-by-step from each Cys residue. For example, in the Zn(II) site IV the first thiolate donor to dissociate is Cys7, followed by Cys24 and then Cys21. The last Cys residue coordinating Zn(II) is Cys5.

Zinc sites IV and II exhibit a dissociation pattern where the Zn(II) ion first dissociates from bridging thiolates (Fig. 1b) followed by the terminal ones. Our SMD simulations show that in general, the terminal thiolates are more nucleophilic than the bridging ones. However, in the case of zinc site III, the first thiolate to dissociate, Cys26, is a terminal one. Figure 8b displays force-extension curves for each metal dissociation process, with each peak representing a Zn−S dissociation event characterized by a particular force. The curves for zinc sites IV and II exhibit a similar pattern, with negligible differences between the magnitude of the force peaks.

Interestingly, the force profile of the zinc site III showed significant differences compared to other sites. The dissociation of Zn−S(Cys26) required the highest force and dominated over the rest of the force peaks. To determine the average rupture force, we calculated histograms from the force-extension curves and fitted them to Gaussian distributions (Fig. 8c). We also recorded the total work done during this process (Fig. 8d). The average rupture force and total work done were remarkably lower for Zn(II) site III, while sites IV and II showed similar values (Table 3). These findings suggest that Zn(II) dissociates first from the zinc site III, as it is bound to site III with lower strength compared to sites IV and II.

**Table 3.** Steered MD simulations for the Zn_7_MT2 system^a^

	Order of Zn–S bond break					
Zn(II) binding site	1	2	3	4	Mean rupture force (kcal/mol)	Total work done (kcal/mol)
IV	Cys7	Cys24	Cys21	Cys5	36 ± 13	411 ± 26
III	Cys26	Cys7	Cys15	Cys13	30 ± 9	341 ± 9
II	Cys24	Cys15	Cys29	Cys19	35 ± 13	412 ± 16

^a^The analysis shown refers to the most populated pathway. In the Supplementary data all of the pathways identified for each zinc site can be found. The nomenclature for the Zn(II) binding site refers to Supplementary Fig. S3.

## Discussion

### Impact of MT2 truncation on metal-to-protein composition

As explained earlier, in this study, we used several truncated and mutated MT2 proteins to investigate more deeply into Zn(II) metalation mechanisms and the thermodynamics of bound Zn(II) ions. Previously collected data, although in part divergent, indicate that the mechanism of Zn(II) (un)binding to MTs has a critical biological impact on protein functioning and whole cellular zinc homeostasis.^1,33,37,46^ The major remaining question is how the affinities of Zn(II) ions in MTs are differentiated by several orders of magnitude having the same coordination environment (ZnCys_4_) and how it affects the biological role of this group of proteins in Zn(II) storing or buffering under various cellular conditions. To shed light on that issue, we focused on two types of protein alterations. In the first, one or two amino acid residues were mutated and Zn(II) binding properties were investigated. In the second one, the β-domain, where loosely bound Zn(II) is localized, was separated from the α-domain (isolated αMT2, βMT2) or combined with non-Zn(II) binding small protein, which by its size mimics the volume of the α-domain. In that case a highly structured fragment (HP-35) of villin-1 was chosen (βMT2-villin).

To date, metallothionein mutants have not been thoroughly characterized in terms of Zn(II) binding and stability of formed metal complexes. The only available reports utilized cadmium metallothionein, and the conclusions may not necessarily be translated to its zinc counterpart in all biochemical or structural aspects. Cysteine mutation to other residues was investigated using monkey MT1.^67^ This study showed that Cys33 does not diminish the stability as after purification and concentration the C33M-MT1 mutant binds 7 Cd(II) mol. eq. On the other hand, point mutations of Cys13 or Cys50 residues to tyrosine in Chinese hamster MT2 resulted in proteins that bind only 6 Cd(II) mol. eq. similarly to C21A-MT2 in this study but its Zn(II) loading was decreased to ∼5.5 Zn(II) mol. eq. (Table 1).^68^ The effect of double C13Y and C50Y residues’ mutation was not additive because a double mutant (C13Y_C50Y-MT2) could also bind 6 Cd(II) ions, indicating that not all Cys residue are critical for decreasing equivalents of bound metal ions.^68^

Mutants of non-metal binding residues have been shown to have some impact on the stability or reactivity of MTs but not their metal composition. Unfortunately, the effects were studied only for Cd(II), not Zn(II) complexes. For instance, Thr5 residue mutation to alanine in human MT3 exhibited distinct metal thiolate activity in EDTA and DTNB reactions, but also lost neuronal inhibitory activity. Thr5 mutation to Ser rescued biochemical and neuronal activity, indicating the importance of the hydroxyl group at this position in the β-domain.^69^ Similarly, Glu23 residue mutation eliminates biological activity in MT3 without affecting metal composition, but increases protein reactivity toward S-nitrosocysteine, suggesting that mutation at Glu23 may alter the NO metabolism and/or affect zinc homeostasis in the brain.^70^ Single or double mutation of Lys30 and Lys31 residues to glutamate or alanine did not affect Cd(II) binding as obtained proteins were able to bind seven metal ions.^71,72^ However, mutation of both lysine residues was observed to reduce the ability of MT to protect yeast transformants against otherwise toxic levels of cadmium. This diminished metal detoxification capacity was due to a decrease in the steady-state level of MT. It showed that at least one charged amino acid must be present in the hinge for the proper expression of MT. It should however be remembered that the conclusions are based on the toxic effect of cadmium on yeast cells expressing Chinese hamster ovary MT2.^71^ Our results, based almost exclusively on Zn(II) composition studies, show that all MT2 mutants besides C21A-MT2 bind 7 Zn(II) mol. eq., indicating that mutated residues do not affect protein composition, but they have an impact on Zn(II) binding thermodynamics and protein reactivity (see the following text). Elimination of the Cys21 residue, which was shown to be the last modified Cys residue in locking the Zn_3_Cys_9_ cluster of the β-domain, significantly impacts Zn(II) binding in MT2.^38^ Independent isolations of Zn(II)-loaded C21A-MT2, under the same conditions as other proteins, resulted in proteins with less than 6 Zn(II) mol. eq. Altogether, this shows that protein loses one Zn(II) ion due to more likely different metal core formation (Zn_2_Cys_8_) and the next one is significantly weakened.

Among other investigations on MTs’ truncation, those based on human MT3 and MT1 hybrid and mutant proteins constructed by Huang *et al*. are worth mentioning. Constructed βMT3-βMT3 and βMT3-αMT1 proteins linked with the KKS linker bound 6 and 7 Cd(II) mol. eq., respectively, as expected. However, the presence of two β-domains in one hybrid protein significantly decreased overall stability, while the second mutant demonstrated Cd(II) binding affinity of the β-domain comparable to that of WT MT3.^73^ These data showed the importance of the KKS linker in modulating the stability of metal–thiolate clusters and conformation of the β-domain through domain–domain interactions, and the influence of protein bioactivity. Another study on MT3 showed that KKS linker mutation to SP caused the loss of neuronal inhibitory activity completely without Cd(II) binding composition change.^72^ This indicates the critical role of the linker in the stability and solvent accessibility of the β-domain required for proper MT3 activity. In this study, we found by connecting the β-domain and villin protein through the KKS linker that some Zn(II) binding properties of the β-domain (see the following text) can be rescued compared to the isolated domain, but the metal composition remains almost the same.

### Impact of MT2 truncations or mutation on Zn(II) affinity and reactivity of Cys residues

Competition of MT2, its domains and mutants with PAR revealed that most of the alanine mutants behaved similarly to WT protein with the exception of the double K30A_K31A mutant and C21A, which resulted in reduced affinity for Zn(II) by ∼0.36 and 0.67 orders of magnitude, respectively. However, a major increase of affinity was observed for the isolated domains. These results indicate that the separation of domains governs the affinity of the zinc sites, rather than particular amino acid residues. This conclusion is further supported by the results from βMT2-villin, resembling the full-length MT2. A final piece of evidence was provided by competition with the fluorogenic sensor ZnAF-2F, which showed that isolated domains form much more stable complexes with Zn(II) than full-length protein. As mentioned earlier, the βMT2-villin hybrid possesses a Zn(II) binding site that is similar to MT2 in terms of its thermodynamics.

Differences in the reactivity of isolated domains compared to a whole protein were also observed in the past by Jiang *et al*., who compared Zn(II) binding properties and reactivity of all Lys residues in domains and full-length MT2 protein.^45^ Interestingly, cumulative properties of the individual domains do not overlap properties of the intact protein, indicating that the two-domain structure of MT2 is important for its interaction with ligands and for controlling reactivity and overall conformation. Indeed this was observed in our previous MS investigations on Zn(II) pathway binding mechanism in human MT2.^38^ The reactivity of partially Zn(II)-loaded MT2 and the isolated β-domain with iodoacetamide indicated significant differences in the localization of labelled residues. In whole protein the Cys residues demonstrating the highest reactivity toward iodoacetamide are those unbound to Zn(II) and specific metalation mechanisms are indicated in both domains. For the isolated β-domain no specificity was observed in Cys-residue labeling besides a decreasing tendency for modification with an increasing number of Zn(II) equivalents bound. This strongly indicated that Zn(II) ions bind to various Cys resides (binding sites) during metalation, suggesting no specific folding mechanism as in a whole system.

To gain a full understanding of metal binding affinities in this report, we also performed a reactivity study of investigated proteins with thiols. These results confirmed that in the isolated domains the cysteine residues are efficiently bound by the Zn(II) due to higher affinity. The reactivity of βMT2 was rescued to MT2 level by its fusion with villin. Similarly as earlier, the alanine mutants showed limited change in reactivity toward DTNB. Due to different metal loading, the C21A mutant provided some of the highest reaction rates, suggesting that some cysteine residues are not involved in the Zn(II) binding or are highly reactive due to significant negative charge and prone to modification. The previous studies on metallothionein mutants, described earlier, either focused only on resistance to Cd(II) or did not characterize. the Zn(II) complex in depth, so we have no point of reference to our data. However, the presence of a low affinity zinc site is in agreement with our previous findings.^38^

### Domains connection in MT2 induces differences in domains folding

In our previous study, we used SMD involving Zn_7_MT2 to investigate the mechanism of protein unfolding.^33^ To achieve this, the C-terminal half of the protein was fixed, while the N-terminal was pulled out at a constant speed. As a result, we observed that protein unfolding was accompanied by Zn(II) dissociation from Zn(II) site IV in the β-domain (Fig. 1b). Interestingly, while using metadynamics simulations to assess the strength of each Zn−S bond in all zinc sites, we discovered a metastable Zn–S(Cys21) bond at site IV and Zn–S(Cys29) bond at site II, which is in contrast with zinc site IV being the weakest site.

In order to address these discrepancies, we conducted an experimental investigation of Zn(II) (un)binding from/to cysteine residues in site IV using the C21A mutant. Our results indicate that the C21A mutation has a negative impact on Zn(II) binding, suggesting that Cys21 is involved in strong interactions with Zn(II). This finding is consistent with our previous metadynamics simulations, which showed a metastable Zn–S (Cys21) bond at site IV, but it contradicts our previous SMD simulations which suggested that zinc site IV was the weakest.^33^ We hypothesized that the discrepancy between the SMD and metadynamics simulations may be due to different molecular mechanisms being studied. In the SMD simulations, we focused on protein unfolding, as described earlier. However, in the metadynamics simulations, we investigated the binding and unbinding of Zn(II) to and from MT2. To test our hypothesis, we performed SMD simulations with a restrained protein backbone to study Zn(II) unbinding from MT2 in the absence of protein unfolding. By pulling Zn(II) out of MT2 at a constant speed while restraining the protein backbone, we were able to exclude any effects that might arise from protein unfolding and focus solely on Zn(II) binding.

It is not possible to determine experimentally whether protein folding is coupled with Zn(II) (un)binding in MTs, as they lack secondary structural elements or aromatic amino acids.^1^ However, using this MD approach, we found that Zn(II) unbinding from zinc site III required less energy than for zinc site IV and site II. Our findings are consistent with both our experimental results presented here and our previous metadynamics simulations, which demonstrated strong interactions between Cys21 and Cys29 with Zn(II) at sites IV and II, respectively. These results suggest that neither site IV nor site II dissociates Zn(II) in the absence of an external force that modulates the protein backbone.

Interestingly, in the crystal structure of rat hepatic MT2, the sidechain of Lys31 interacts with the mainchain of the β-domain, forming two hydrogen bonds with the carbonyl oxygen of Cys21 and Cys19 (Supplementary Fig. S3). The removal of this residue results in a decrease of Zn(II) affinity, as does the removal of Lys20, which is positioned between both cysteine residues. Our SMD results correlate well with the experimentally obtained evidence indicating that Cys21 is tightly bound to Zn(II) at site IV. Removal of the Lys31 residue has no significant impact on site IV due to tight interaction of Zn(II) with Cys21 but affects the stability of zinc site III containing Cys19 (Supplementary Fig. S3).

Each lysine residue mutation studied here has an impact on the affinity of the weakest zinc site due to either a specific interaction with the weakest site or an overall loss of positive charge required to compensate for the negative charge of the Zn_3_Cys_9_ cluster. As a result, the mutation(s) partially affects the electrostatic interaction network required for differentiation of Zn(II) ions’ affinities. The physical separation of both domains must have the most critical effect on the electrostatic network in the β-domain of the whole protein, which collapses in the isolated domain. As a consequence, the zinc sites of the isolated domain demonstrate a significant difference in thermodynamics and coordination dynamics compared to the whole protein. This network is partially rescued in the β-MT-villin domain, which demonstrates metal binding similarities to the WT-MT2.

The data obtained in this study and the earlier discussion indicate that the mechanism of Zn(II) metalation in the β-domain significantly changes from the isolated domain to the whole protein. The three Zn(II) ions in the highly flexible isolated β-domain demonstrate similar affinity for their sites. Moreover, there is no specific binding pathway for a particular Zn(II) ion because they bind to various cysteine residues, forming a heterogenic system, which has been confirmed by MS.^38^ This results in significant likeness of Zn(II) affinities, which fall in the range of low picomoles (Table 1). This value is typical for tetrathiolate coordination zinc sites in peptides or protein fragments that demonstrate high flexibility.^41,44,48,60,74,75^ High flexibility of the isolated β-domain was also observed for the Cd(II)-loaded β-domain, for which it was impossible to record ^113^Cd or ^111^Cd resonances, in contrast to the α-domain, which is much more rigid and structured.^76–78^ When the β-domain becomes less flexible due to connection to the α-domain—with a decrease of degrees of freedom—the metalation process changes. It should be noted that the loss of freedom is also translated to the α-domain. A previous study demonstrated that Zn(II), in contrast to Cd(II), binds to both domains in such a way that a high number of Cys residues are coordinated, forming a weakly or non-clustered system.^32,33,79^ In the next steps, additional Zn(II) ions join previously formed cores and complete first the α- then the β-cluster. This is not without significance for the binding affinity of particular sites. The first four sites bind Zn(II) with the highest affinity in the low picomole range, while the others bind with progressively less affinity. The β-MT2-villin system is significantly different from the whole, two-domain protein, but the close proximity of the β-domain to villin and the loss of degrees of freedom change the Zn(II) folding mechanism compared to the isolated domain, making it start to resemble that of the whole protein. This is observed at least by the differentiation of affinities of the three Zn(II) ions indicated in the PAR and ZnAF-2F experiment (Table 2). This altogether indicates that Zn(II) binding to MT2 occurs through an entropically driven mechanism, while Cd(II) binding, which involves the formation of specific clusters, occurs through an enthalpically driven pathway.^1,20,32,33,79^

### Biological and practical significance of MT domains’ connection

Metallothioneins play fundamental roles in the metabolism of zinc and copper in mammals, but the connection between these two metal ions remains incompletely understood.^1,3^ It is important to note that besides MTs there are other proteins involved in zinc homeostasis such as zinc transporters, both importers and exporters (ZnT, Zip), as well as recently discovered escort protein(s).^72,73,80–82^ MTs were believed for many years to bind all Zn(II) ions with the same, undistinguished affinity, with *K*_d_^av^ depending on the study varying from sub-picomoles to a few picomoles (10^−13^–10^−^^11^ M).^22,34–37,59,83^ It is worth noting that most of the structural and catalytic zinc sites are bound in this range of affinities; therefore the role of MTs in the handling of Zn(II) in the cell remained a subject of numerous discussions.^84,85^ Two important findings have changed the point of view on Zn(II)-to-MT affinity and its role in cellular zinc metabolism. The first one is related to the development of sensitive fluorescent zinc probes that have been used to determine free Zn(II) (also called labile or exchangeable zinc) concentration and its fluctuations.^38,86,87^ After two decades of their application for cells, tissues and living organisms it became clear that its concentration varies from few nM to hundreds of picomoles in most cases (10^−8^–10^−^^10^ M); however, lower and higher concentrations were reported, for instance in cellular compartments.^44,88,89^ Based on that, an important question arose: how MTs may participate in controlling free Zn(II) if their affinity is in the low picomolar range. Then another finding shed light on this issue. Using FluoZin-3 and RhodZin-3 it was shown that human MT2 binds seven Zn(II) in the range of nano- to picomolar, exactly matching the reported range of free Zn(II).^62,63,90^ According to that, four Zn(II) ions are bound with high affinity (*K*_d4-7_ ∼10^−^^12^ M), two ions are bound with moderate affinity (*K*_d2-3_ ∼10^−^^10^ M) and one site with nanomolar affinity (*K*_d1_ ∼10^−^^8^ M).^38^ Importantly, differentiation of these zinc sites has been confirmed using numerous competing experiments with catalytic, structural and regulatory zinc sites; however, it is still the subject of scientific discussion.^39,40–42,44,45^ In this study, we therefore focused on shedding new light on the mechanism of such differentiation of thermodynamics. From the chemical point of view it was unclear how the same tetrathiolate zinc sites may differ in their affinity even by up to four orders of magnitude. This value corresponds to ∼5.5 kcal/mol difference and represents a significant energetic cost resulting from the metalation pathway and associated electrostatic interactions occurring in both domains, especially in the β-domain.^1^ Gradual formation of zinc sites (centers) with non-bridging cysteine residues and subsequent closure of domains together with the formation of an internal network of electrostatic interactions is the key thermodynamic factor determining the different affinity of Zn(II) ions in MTs.^32,33^

**Fig. 9:**
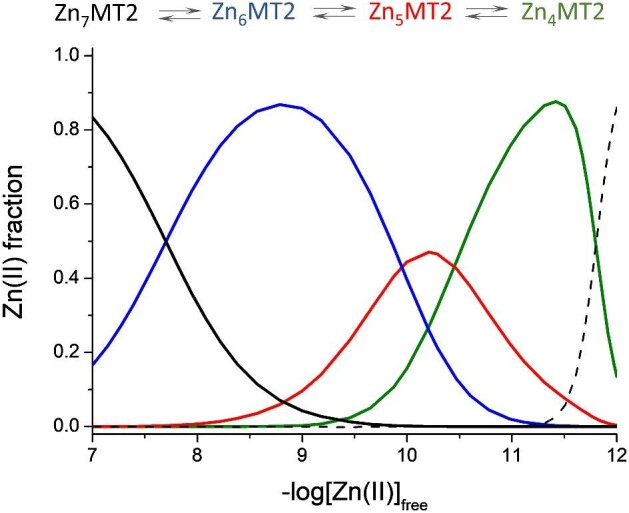
Zn(II)-MT2 species distribution as a function of free Zn(II) changes. The plot was prepared based on previously published stability constants.^38^ Dashed line indicates metal-free protein (thionein) occurring at very low free Zn(II) concentration.

Differential stability of zinc sites in MTs has a significant impact on their biological function, such as cellular free zinc buffering. Occupation of numerous sites with various affinities results in coexistence of variously metalated Zn(II)-MT2 species under physiological conditions, such as Zn_7_MT2, Zn_6_MT2, Zn_5_MT2, or even Zn_4_MT2.^1,38,39^ These species are formed with decreasing free Zn(II) concentration from ∼10^−^^8^ M to 10^−^^11^ M found in eukaryotic cells (Fig. 9). It means that depending on zinc status—zinc surplus, normal or deficiency—various Zn_7-x_MT species predominate and play the role of a Zn(II) donor or acceptor (buffer components). For instance, it has been shown that Zn_7_MT, Zn_6_MT, and Zn_5_MT2 but not Zn_4_MT may serve as a donor for depleted sorbitol dehydrogenase while only Zn_7_MT and Zn_6_MT may (partially) inhibit phosphate tyrosine protein 1B (PTP 1B).^39^ In the case of structural zinc sites, all Zn_7_MT2-Zn_4_MT2 may support the most stable CCHH zinc finger domains, while in the case of naturally altered zinc finger domains this donation is limited to particular species. For instance, the zinc finger ZNF442 with an altered last His residue in CCHH motifs can be filled by Zn_5_MT2 -Zn_7_MT2 species, while the ZScan20 zinc finger with an altered second Cys residue is filled only by Zn_7_MT2 species. Remaining MT2 forms act as an acceptor of Zn(II) from that domain.^41^

Finally, an important practical issue is related to the thermodynamic effects of domains’ connection in metallothioneins. Isolated domains are frequently used in biochemical and biophysical studies as models of whole MT proteins.^1,4^ Due to lower size they are easier to synthesize and study. Although they together bind seven Zn(II) ions, not all effects observed in that case can be translated to the whole protein. One such characteristic feature is the folding, observable especially in the case of Zn(II). As discussed earlier, Zn(II) binds at a low Zn(II)-to-protein molar ratio to both domains with low picomolar affinities and then filled clusters with sites of lower affinities. Investigation of separate domains, although interesting from the scientific point of view, does not explain effects observable in the whole domain.^45,46^ In the last two decades, MS has become a powerful technique in studying metalation/folding of metalloproteins.^21,23,27–29,32,33,37,91,92^ It should be underlined that some conclusions might be inappropriate and the effects must be studied with caution. However, as mentioned earlier, this mostly related to Zn(II) and more likely to Co(II).^93,94^ Cd(II) and Cu(I) bind more specifically to particular domains demonstrating enthalpy-favored folding.^1,83^ Moreover, the present data show that any type of alteration of the MT2 sequence has an impact on protein properties. Some mutations do not change the metal composition but impact Zn(II)-to-protein affinity. Others are critical and cause disruption of the metal cluster(s) and major changes in stability and protein folding mechanisms.

## Conclusions

The present results shed new light on our knowledge of the thermodynamics of Zn(II) binding to metallothioneins and its consequences. It should be underlined that mammalian MTs are zinc and copper proteins, and their investigation with Cd(II) as a convenient spectroscopic model does not necessarily express the properties of the zinc counterpart. By conducting stability and reactivity investigations on WT MT2, its isolated domains, and several mutants, we observed that any alteration of the sequence or structure of the intact protein impacts the Zn(II) binding properties of MTs. Especially important is the proximity/connection of the α- and β-domains, which impacts Zn(II) binding pathways and binding affinity. Separation of the domains increases the degrees of freedom, making domains more dynamic from the coordination point of view. It is caused by the disruption of intra- and interdomain electrostatic interactions. The connection of rigid and small villin-1 to the β-domain partially rescued the properties of the β-domain, confirming the importance of limiting intradomain interactions and degrees of freedom. Application of SMD demonstrated that position III in WT-MT2 is a weak Zn(II) binding site. Overall, close proximity of domains has a critical impact on the differentiation stability of zinc sites in MTs, their metalation mechanism, and reactivity. It has a critical impact on the role of MTs in the cellular environment where they function not only as a sponge of Zn(II) but also as a buffering system of Zn(II) ions, keeping them in the right concentrations or right fluctuations. Therefore, the presence of partially metalated MT species is critical for regulation of the activity of many zinc proteins, their complexes, and dependent cellular pathways.

## Experimental section

### Materials

Bacterial cells were cultured using: tryptone, yeast extract, Luria-Bertani (LB) broth, agar, ampicillin, chloramphenicol (BioShop); glycerol, KH_2_PO_4_, K_2_HPO_4_, isopropyl-D-1-thiogalactopyranoside (IPTG) (Carl Roth); and antifoam 204 (Sigma-Aldrich). Chitin resin was bought from New England BioLabs. All oligonucleotides and other chemicals (buffers, salts) were purchased from Sigma-Aldrich. Buffers’ pH was adjusted using either hydrochloric acid (HCl, NORMATOM) or ultra-pure sodium hydroxide purchased from VWR. The concentration of metal ion salt stock solutions was 0.05 M and was confirmed by a representative series of ICP–MS measurements. All pH buffers were treated with Chelex 100 resin to eliminate trace metal ion contamination.

### Preparation of protein expression vectors

The pTYB21 vector belonging to the Intein Mediated Purification with an Affinity Chitin-binding Tag system (IMPACT, New England BioLabs) was chosen for expression in bacterial cells. The codon optimized sequence for the β-villin gene was synthesized by ATG: Biosynthetics and was delivered in the pUC vector, which was subcloned in the pTYB21 vector. Human MT2 (Addgene plasmid no. #105693) plasmid was used as a template for amplification of β-domain and α-domain gene constructs. The β-villin, β-domain, and α-domain genes were PCR amplified using appropriate primers. Sequences of primers along with PCR conditions can be found in Supplementary data. PCR amplicons and the empty pTYB21 vector were then double digested in parallel with SapI and EcoRV (PstI for α-domain) restriction enzymes and ligated using T4 DNA ligase. All enzymes were purchased from Thermo Fisher Scientific. The ligation mixture was then directly used for transformation of competent *Escherichia coli* DH5α cells. The plasmids were isolated using a miniprep plasmid isolation kit (Syngene) and their correctness was confirmed by colony PCR, restriction digestion and DNA sequencing (Microsynth AG, Germany). The human MT2 (Addgene plasmid ID 105693) plasmid was also used as a template to prepare selected mutants: S6A-MT2, K20A-MT2, C21A-MT2, K30A-MT2, K31A-MT2, K30AK31A-MT2. Plasmids have been deposited in Addgene, plasmids no. #105699, #105698, #105697, #105694, #105695, and #105696, respectively. Details about the mutagenesis procedure as well as a list of used primers can be found in Supplementary data. Presence of desired mutations was confirmed by sequencing.

### Expression and metal-free protein purification

Human MT2, its mutants, domains and fusions were expressed following our previously established protocol.^46^ Briefly, the positive clones were transformed into BL21 (DE3) RIL *E. coli* cells and a primary culture was inoculated in LB medium at 37°C overnight with constant shaking at 180 rpm. The secondary culture was inoculated in a rich full culture medium (1.1% tryptone, 2.2% yeast extract, 0.45% glycerol, 1.3% K_2_HPO_4_, 0.38% KH_2_PO_4_) by adding 1% (v/v) primary culture and grown at 37°C until OD_600_ reached 0.6–0.8. Cells were induced with 0.1 mM IPTG and incubated overnight at 20°C with constant shaking at 180 rpm. The cell pellet was collected by centrifugation (4000 ×*g* for 10 min, 4°C), resuspended in 50 ml of ice cold buffer A (20 mM 4-(2-hydroxyethyl)piperazine-1-ethanesulfonic acid (HEPES), pH 8.0, 500 mM NaCl, 1 mM TCEP), and sonicated for 20 min (5 s “on” and 5 s “off”) followed by centrifugation (20 000 ×*g* for 15 min, 4°C). The supernatant was incubated with 20 ml of chitin resin (pre-equilibrated in buffer A) overnight for protein binding. The next day, resin was washed four to five times with 50 ml of buffer A. Cleavage was induced by adding 100 mM DTT.^51^ The resin was incubated for 48 h at room temperature on a rocking shaker. Eluted solution was acidified to pH ∼2.5 with 7% HCl and concentrated using Amicon Ultra-4 Centrifugal Filter Units with a membrane cut-off of 3 kDa (Merck Millipore, USA). It was subsequently purified on a size exclusion chromatography SEC-70 gel filtration column (Bio-Rad) equilibrated with 10 mM HCl.^38^ The identity of apo-MT proteins (thionein) was confirmed by ESI-MS (API 2000, Applied Biosystems). The concentration of thiols was determined spectrophotometrically (Jasco V-650 or V-630) using Ellman's reagent—DTNB.^57^ Ellman's method involves modification of free thiols in proteins by DTNB and subsequent release of TNB^−^, which is detected spectrophotometrically at 412 nm. DTNB reagent was prepared fresh before use in the concentration of 1 mM in 50 mM Na^++^HEPES, pH 7.4. Due to MT's ability to specifically bind biarsenical dyes, it was possible to monitor the expression and purification of MT2 and its constructs on SDS-PAGE gels. Prior to separation the protein samples were denatured and incubated for 2 h at room temperature with 5 µM F4-FlAsH-EDT_2_ in the presence of 200 µM TCEP, similarly as described before.^52,53,95^ After the electrophoresis the bands were visualized with UV light.

### Reconstitution of zinc proteins

Purified thioneins (apo-MTs) were immediately mixed with excess ZnSO_4_ and 1 mM TCEP under a nitrogen blanket. A 10 molar excess of ZnSO_4_ was added to apo-MT2A, a 6 molar excess to the apo-α-domain, and a 4.5 molar excess to the apo-β-domain and apo-βMT2-villin. The pH of the solution was then adjusted to 8.6 with a 1 M Tris solution. Holo-MTs were then concentrated using 3 kDa Amicon Ultra-4 Centrifugal Filter Units (Merck Millipore). Subsequently, they were purified on a SEC-70 gel filtration column (Bio-Rad) equilibrated with 20 mM Tris-HCl, pH 8.6.

### Determination of Zn(II) and thiol(ate) concentrations with PAR and DTNB

Total amount of Zn(II) bound to holo-MTs was determined using the PAR assay.^58^ When present in excess, PAR binds Zn(II) to form Zn(PAR)_2_, which is detected spectrophotometrically at 492 nm. A stock solution of 20 mM PAR was prepared in DMSO for long storage at room temperature. The stock was diluted before use to 100 µM concentration in 50 mM Na^++^HEPES, pH 7.4 and 1 mM DTNB solution. DTNB was added to modify free thiols and prevent Zn(II) rebinding. Concentrations of thiols in holo-MTs were determined spectrophotometrically using 1 mM EDTA in DTNB solution.^57^ EDTA is a strong metal chelator that releases metal ions bound to MTs and enables modification of free thiols by DTNB.

### ICP–AES measurements

ICP–AES measurements were done in the Mass Spectrometry Laboratory at the Life Sciences University in Wrocław (Poland). Samples were analysed by ICP (ICP-AES iCAP 7400, Thermo Scientific) to confirm the spectrophotometric results. Prior to analysis protein samples were diluted with 0.5 M ultra pure nitric acid. The final concentration of MT2 and its variants was in the range 10–20 µM. Control samples with 20 mM Tris-HCl, pH 8.6 were also prepared. Calculation of Zn(II) molar concentration accounted for increased density of the solvent.

### Spectrophotometric metal-to-protein titrations

The titration of metal-free MT2, βMT2, and βMT2-villin with ZnSO_4_ and CdSO_4_ was monitored spectrophotometrically (Jasco V-650) at 25°C in the 200–300 nm UV range. 1 µM MT2A or 2 µM βMT2/βMT2-villin was titrated with 500 µM ZnSO_4_ or CdSO_4_ in chelexed 50 mM borate buffer (100 mM NaClO_4_, pH 7.4) and 100 µM TCEP under anaerobic conditions. A total of 50 mM borate buffer (100 mM NaClO_4_, pH 7.4) with 100 µM TCEP was blanked in a 1 cm quartz cuvette followed by mixing with an appropriate concentration of apo-MT solution prepared in 10 mM HCl. The resultant solution was then titrated with subsequent 0.25–1 molar equivalents of 500 µM ZnSO_4_ or CdSO_4_. One accumulation was recorded using a 2 nm band width, a 200 nm per min scanning speed and a 1.0 nm data pitch. Spectra were averaged from the following two accumulations.

### Spectrophotometric competition with PAR

Spectroscopic observation of competition of MT2, its domains, fusion and mutants with PAR was performed in 50 mM Na^++^HEPES buffer, pH 7.4, with 0.1 M NaCl and 200 µM TCEP. The concentration of proteins was 1.7 µM and of PAR was 200 µM PAR. Kinetics of Zn(II) transfer to PAR were monitored at 492 nm and then recalculated to Zn(II) mole equivalents using the molar absorption coefficient 71 500 M^−1^ cm^−1^. Calculation of the K_d_^av^ values is outlined in the results and it follows our previously published method.^57^

### Fluorimetric competition with ZnAF-2F

Fluorometric studies were performed on a Fluoromax-4 (Horiba Scientific, USA) with Temperature Controller 350B (Newport, USA) at 25°C using single-use 10 mm light path polystyrene cuvettes (Sarstedt, Germany). Emission signals were collected on 1 nm slit settings (both width and length) at 25°C using wavelengths 492 nm for excitation and 516 nm for emission (ZnAF-2F measurements).^64^ To determine the weakest bound Zn(II), 0.5 µM protein was incubated with 0.05–3 µM ZnAF-2F for 90 min in the dark at 25°C. Maximum fluorescence was measured by saturating ZnAF-2F with an excess of Zn(II) (*F*_max_). Minimum fluorescence was measured by adding the metal chelator EDTA in excess (*F*_min_). Controls were performed by addition of 50 µM oxidation agents (DTNB, NPSC), to obtain the values of fluorescence corresponding to all cysteine residues oxidized, thus all Zn(II) released from MT.

### Oxidation of MTs and domains by DTNB

Oxidation of MT2, its domains and mutants (1.7 µM) was performed with 2 µM DTNB in 50 mM Na^++^HEPES buffer, pH 7.4, 0.1 M NaCl. In this study MT2 and its mutants and domains were used at 1.7 µM concentrations, while DTNB was applied in a very slight excess of 2 µM. Depending on the protein used, the kinetic reaction was carried out for between 50 and 120 min. Supplementary Fig. S2 presents examples of DTNB reduction kinetics. Under the applied conditions for all cases, besides the α-domain, kinetics were monophasic and the absorbance increase fitted the first-order kinetic rate equation to determine *k*_obs_ values.^38^ Figure 7a presents the comparison of obtained *k*_obs_.

### Computational studies

#### System set-up

The X-ray structure PDB ID 4MT2 was the initial structure for the computational studies.^13^ The Zn_7_MT2 system was constructed by replacing the four Cd(II) by Zn(II). The protonation states of the side chains at pH 7.0 were assigned using PROPKA, except for the Cys residues that were deprotonated. The AMBER FF19SB force field and recently published cysteine-Zn(II) force field parameters were used to model the protein and the Cys residues.^96,97^

#### SMD simulations

Constant-speed SMD simulations were used to study the Zn(II)-unbinding of Zn_7_MT2. The protein was solvated in an 8 Å cubic box with TIP3P water molecules and NaCl was added to achieve neutrality. The systems were equilibrated in four steps. First, 10 000 steps of steepest descent minimization were applied to the system. Second, the system was heated up from 0 to 300 K in the NVT ensemble using the Langevin thermostat with a damping coefficient of 1 ps^−1^. Third, the system was equilibrated at constant pressure and temperature (NPT) for 100 ns. The pressure was kept at 1 atm and the temperature at 300 K using Berendsen weak coupling. In the last step, 100 ns were run using the Parinello-Rahman barostat and the Nosé-Hoover thermostat. The particle mesh Ewald algorithm was used to evaluate electrostatic interactions using a cut-off of 8 Å. The LINCS algorithm was used to constraint bonds only involving hydrogen atoms to allow the use of a 2 fs time step. Then, 10 independent SMD runs at constant speed were performed for each Zn(II) site, by using as a collective variable (CV) the distance between the Zn(II) and the center of mass of the initially four Cys residues to where it is coordinated. To avoid distortions in the protein backbone due to the force applied, 10 kcal mol^−1^ positional restraints were applied to all of the CA atoms.

Four spring constants of 10, 20, 50, and 100 kcal mol^−1^ and three velocities (50, 100, 150 Å·ns^−1^) were tested. A force constant of 100 kcal·mol^−1^ and pulling speed of 100 Å·ns^−1^ were chosen, as this did not introduce any artefacts and allowed the fastest regime. The CV was pulled from 0 Å to 27 Å in 135 000 steps of 2 fs. The computations were performed with GROMACS 2018.4 in combination with the PLUMED plugin.^98,99^ To identify Zn−S dissociation pathways, the contact number (CN) between Zn(II) and each Cys sulfur was determined using the CN defined as:


(9) \begin{eqnarray*}
C\ {N}_{Zn - S} = \mathop \sum \limits_{i \in A} \mathop \sum \limits_{i \in B} {s}_{ij}\
\end{eqnarray*}

where *A* is the Zn(II) ion, *B* corresponds to the Cys(S) residue, and s_ij_ is a switching function. The switching function is defined as:


(10) \begin{eqnarray*}{{\boldsymbol{s}}}_{{\boldsymbol{ij}}} = \frac{{1 - {{\left. {\left( {\frac{{{{\boldsymbol{r}}}_{{\boldsymbol{ij}}}}}{{{{\boldsymbol{r}}}_0}}} \right.} \right)}}^{\boldsymbol{n}}}}{{1 - {{\left. {\left( {\frac{{{{\boldsymbol{r}}}_{{\boldsymbol{ij}}} - }}{{{{\boldsymbol{r}}}_0}}} \right.} \right)}}^{\boldsymbol{m}}}} \end{eqnarray*}

where *n* = 8 and *m* = 12 and they define the steepness of the switching function, and *r*_0_ = 2.8 Å, which defines the cut-off to where the interactions between Zn(II) and the sulfur donor are calculated.

## Supplementary Material

mfad029_Supplemental_File

## Data Availability

The data underlying this article are available in the article and in its online supplementary material.

## References

[1] Krężel A., Maret W., The Bioinorganic Chemistry of Mammalian Metallothioneins, Chem. Rev., 2021, 121 (23), 14594–14648. 10.1021/acs.chemrev.1c00371 34652893

[2] Nordberg M., Nordberg G. F., Metallothioneins: Historical Development and Overview, Met. Ions Life Sci., 2009, 5, 1–29.

[3] Krężel A., Maret W., The Functions of Metamorphic Metallothioneins in Zinc and Copper Metabolism, IJMS, 2017, 18 (6), 1237–1257. 10.3390/ijms18061237 28598392 PMC5486060

[4] Blindauer C. A., Leszczyszyn O. I., Metallothioneins: Unparalleled Diversity in Structures and Functions for Metal Ion Homeostasis and More, Nat. Prod. Rep., 2010, 27 (5), 720–741. 10.1039/b906685n 20442962

[5] Babula P., Masarik M., Adam V., Eckschlager T., Stiborova M., Trnkova L., Skutkova H., Provaznik I., Hubalek J., Kizek R., Mammalian Metallothioneins: Properties and Functions, Metallomics, 2012, 4 (8), 739–750. 10.1039/c2mt20081c 22791193

[6] Apostolova M. D., Ivanova I. A., Cherian M. G., Metallothionein and Apoptosis during Differentiation of Myoblasts to Myotubes: Protection against Free Radical Toxicity, Toxicol. Appl. Pharmacol., 1999, 159 (3), 175–184. 10.1006/taap.1999.8755 10486304

[7] Ye B., Maret W., Vallee B. L., Zinc Metallothionein Imported into Liver Mitochondria Modulates Respiration, Proc. Natl. Acad. Sci. U.S.A., 2001, 98 (5), 2317–2322. 10.1073/pnas.041619198 11226237 PMC30136

[8] Krizkova S., Kepinska M., Emri G., Rodrigo M. A., Tmejova K., Nerudova D., Kizek R., Adam V., Microarray Analysis of Metallothioneins in Human Diseases—a Review, J. Pharm. Biomed. Anal., 2016, 117, 464–473. 10.1016/j.jpba.2015.09.031 26454339

[9] Uchida Y., Takio K., Titani K., Ihara Y., Tomonaga M., The Growth Inhibitory Factor That Is Deficient in the Alzheimer's Disease Brain Is a 68 Amino Acid Metallothionein-Like Protein, Neuron, 1991, 7 (2), 337–347. 10.1016/0896-6273(91)90272-2 1873033

[10] Quaife C. J., Findley C. D., Erickson J. C., Froelick G. J., Kelly E. J., Zambrowicz B. P., Palmiter R. D., Induction of a New Metallothionein Isoform (MT-IV) Occurs during Differentiation of Stratified Squamous Epithelia, Biochemistry, 1994, 33 (23), 7250–7259. 10.1021/bi00189a029 8003488

[11] Si M., Lang J., The Roles of Metallothioneins in Carcinogenesis, J. Hematol. Oncol., 2018, 11 (1), 107–127. 10.1186/s13045-018-0645-x 30139373 PMC6108115

[12] Merlos Rodrigo M. A., Jimenez Jimemez A. M., Haddad Y., Bodoor K., Adam P., Krizkova S., Heger Z., Adam V.., Metallothionein Isoforms as Double Agents – Their Roles in Carcinogenesis, Cancer Progression and Chemoresistance, Drug Resist. Updat., 2020, 52, 100691. 10.1016/j.drup.2020.100691 32615524

[13] Robbins A. H., McRee D. E., Williamson M., Collett S. A., Xuong N. H., Furey W. F., Wang B. C., Stout C. D., Refined Crystal Structure of Cd, Zn Metallothionein at 2.0 A Resolution, J. Mol. Biol., 1991, 221, 1269–1293. 1942051

[14] Arseniev A., Schultze P., Wörgötter E., Braun W., Wagner G., Vašák M., Kägi J. H., Wüthrich K., Three-Dimensional Structure of Rabbit Liver [Cd_7_]Metallothionein-2A in Aqueous Solution Determined by Nuclear Magnetic Resonance, J. Mol. Biol., 1988, 201 (3), 637–657. 10.1016/0022-2836(88)90644-4 3418714

[15] Otvos J. D., Armitage I. M., Structure of the Metal Clusters in Rabbit Liver Metallothionein, Proc. Natl. Acad. Sci. U.S.A., 1980, 77 (12), 7094. 10.1073/pnas.77.12.7094 6938956 PMC350447

[16] Messerle B., Schäffer A., Vašák M., Kägi J. H., Wüthrich K., Three-Dimensional Structure of Human [^113^Cd_7_]Metallothionein-2 in Solution Determined by Nuclear Magnetic Resonance Spectroscopy, J. Mol. Biol., 1990, 214 (3), 765. 10.1016/0022-2836(90)90291-S 2388267

[17] Wang H., Zhang Q., Cai B., Li H., Sze K. H., Huang Z. X., Wu H. M., Sun H., Solution Structure and Dynamics of Human Metallothionein-3 (MT-3), FEBS Lett, 2006, 580 (3), 795. 10.1016/j.febslet.2005.12.099 16413543

[18] Braun W., Vašák M., Robbins A. H., Stout C. D., Wagner G., Kägi J. H., Wüthrich K., Comparison of the NMR Solution Structure and the X-Ray Crystal Structure of Rat Metallothionein-2, Proc. Natl. Acad. Sci. U.S.A., 1992, 89 (21), 10124. 10.1073/pnas.89.21.10124 1438200 PMC50290

[19] Nielson K. D., Winge D. R., Independence of the Domains of Metallothionein in Metal Binding, J. Biol. Chem., 1985, 260 (15), 8698. 10.1016/S0021-9258(17)39405-X 4019449

[20] Stillman M. J., Cai W., Żelazowski A. J., Cadmium Binding to Metallothioneins. Domain Specificity in Reactions of Alpha and Beta Fragments, Apometallothionein, and Zinc Metallothionein with Cd^2+^, J. Biol. Chem., 1987, 262 (10), 4538. 10.1016/S0021-9258(18)61226-8 3558354

[21] Dong S., Shirzadeh M., Fan L., Laganowsky A., Russell D. H., Ag^+^ Ion Binding to Human Metallothionein-2A Is Cooperative and Domain Specific, Anal. Chem., 2020, 92 (13), 8923. 10.1021/acs.analchem.0c00829 32515580 PMC8114364

[22] Vašák M., Overnell J., Good M., Spectroscopic and Chemical Approaches to the Study of Metal-Thiolate Clusters in Metallothionein (MT), Experientia. Suppl., 1987, 52, 179–189. 10.1007/978-3-0348-6784-9_11 2822462

[23] Gehrig P. M., You C., Dallinger R., Gruber C., Brouwer M., Kägi J. H., Hunziker P. E., Electrospray Ionization Mass Spectrometry of Zinc, Cadmium, and Copper Metallothioneins: Evidence for Metal-Binding Cooperativity, Protein Sci., 2000, 9 (2), 395. 10.1110/ps.9.2.395 10716192 PMC2144553

[24] Stillman M. J., Law A. Y., Cai W. H., Żelazowski A., Information on Metal Binding Properties of Metallothioneins from Optical Spectroscopy, Experientia. Suppl., 1987, 52, 203–211. 10.1007/978-3-0348-6784-9_13 2959506

[25] Vašák M., Kägi J. H. R. in Metal Ions in Biological Systems, ed. Sigel H., Dekker Marcel, New York, NY, 1983;15, 213–273.

[26] Munoz A., Rodriguez A. R., Electrochemical Behavior of Metallothioneins and Related Molecules. Part III: Metallothionein, Electroanalysis, 1995, 7 (7), 674. 10.1002/elan.1140070715

[27] Yu X., Wojciechowski M., Fenselau C., Assessment of Metals in Reconstituted Metallothioneins by Electrospray Mass Spectrometry, Anal. Chem., 1993, 65 (10), 1355. 10.1021/ac00058a010 8517548

[28] Chen S. H., Russell W. K., Russell D. H., Combining Chemical Labeling, Bottom-Up and Top-Down Ion-Mobility Mass Spectrometry to Identify Metal-Binding Sites of Partially Metalated Metallothionein, Anal. Chem., 2013, 85 (6), 3229. 10.1021/ac303522h 23421923

[29] Peris-Díaz M. D., Guran R., Zitka O., Adam V., Krężel A., Metal- and Affinity-Specific Dual Labeling of Cysteine-Rich Proteins for Identification of Metal-Binding Sites, Anal. Chem., 2020, 92 (19), 12950–12958. 10.1021/acs.analchem.0c01604 32786475 PMC7547867

[30] Boulanger Y., Goodman C. M., Forte C. P., Fesik C. W., Armitage I. M., Model for Mammalian Metiallothionein Structure, Proc. Natl. Acad. Sci. U.S.A., 1983, 80 (6), 1501. 10.1073/pnas.80.6.1501 6572910 PMC393629

[31] Zangger K., Armitage I. M., Dynamics of Interdomain and Intermolecular Interactions in Mammalian Metallothioneins, J. Inorg. Biochem., 2002, 88 (2), 135. 10.1016/S0162-0134(01)00379-8 11803034

[32] Peris-Díaz M. D., Barkhanskiy A., Liggett E., Barran P., Krężel A., Ion Mobility Mass Spectrometry and Molecular Dynamics Simulations Unravel the Conformational Stability and Coordination Dynamics of Human Metallothionein-2 Species, Chem. Commun., 2023, 59 (30), 4471. 10.1039/D2CC06559B PMC10089061 36960761

[33] Peris-Díaz M. D., Guran R., Domene C., de los Rios V., Zitka O., Adam V., Krężel A., An Integrated Mass Spectrometry and Molecular Dynamics Simulations Approach Reveals the Spatial Organization Impact of Metal-Binding Sites on the Stability of Metal-Depleted Metallothionein-2 Species, J. Am. Chem. Soc., 2021, 143 (40), 16486–16501. 10.1021/jacs.1c05495 34477370 PMC8517974

[34] Otvos J. D., Petering D. H., Shaw C. F., Structure-Reactivity Relationships of Metallothionein, a Unique Metal-Binding Protein, Comments Inorg. Chem., 1989, 9 (1), 1–35. 10.1080/02603598908035801

[35] Namdarghanbari M. A., Meeusen J., Bachowski G., Giebel N., Johnson J., Petering D. H., Reaction of the Zinc Sensor FluoZin-3 with Zn_7_-Metallothionein: Inquiry into the Existence of a Proposed Weak Binding Site, J. Inorg. Biochem., 2010, 104 (3), 224–231. 10.1016/j.jinorgbio.2009.11.003 20007001 PMC2935270

[36] Hasler D. W., Jensen L. T., Zerbe O., Winge D. T., Vašák M., Effect of the Two Conserved Prolines of Human Growth Inhibitory Factor (Metallothionein-3) on its Biological Activity and Structure Fluctuations: Comparison with Mutant Protein, Biochemistry, 2000, 39 (47), 14567–14575. 10.1021/bi001569f 11087412

[37] Pinter T. B. J., Stillman M. J., The Zinc Balance: Competitive Zinc Metalation of Carbonic Anhydrase and Metallothionein 1A, Biochemistry, 2014, 53 (39), 6276–6285. 10.1021/bi5008673 25208334

[38] Krężel A., Maret W., Dual Nanomolar and Picomolar Zn(II) Binding Properties of Metallothionein, J. Am. Chem. Soc., 2007, 129 (35), 10911. 10.1021/ja071979s 17696343

[39] Krężel A., Maret W., Thionein/Metallothionein Control Zn(II) Availability and the Activity of Enzymes, J. Biol. Inorg. Chem., 2008, 13 (3), 401. 10.1007/s00775-007-0330-y 18074158

[40] Zeng J., Vallee B. L., Kägi J. H., Zinc Transfer from Transcription Factor IIIA Fingers to Thionein Clusters, Proc. Natl. Acad. Sci. U.S.A., 1991, 88 (22), 9984. 10.1073/pnas.88.22.9984 1835092 PMC52851

[41] Kluska K., Adamczyk J., Krężel A., Metal Binding Properties of Zinc Fingers with a Naturally Altered Metal Binding Site, Metallomics, 2018, 10 (2), 248–263. 10.1039/C7MT00256D 29230465

[42] Bell S. G., Vallee B. L., The Metallothionein/Thionein System: an Oxidoreductive Metabolic Zinc Link, Chem. Bio. Chem,. 2009, 10 (1), 55. 10.1002/cbic.200800511 19089881

[43] Kochańczyk T., Jakimowicz P., Krężel A., Femtomolar Zn(II) Affinity of Minimal Zinc Hook Peptides—a Promising Small Tag for Protein Engineering, Chem. Commun., 2013, 49 (13), 1312. 10.1039/c2cc38174e 23303248

[44] Kocyła A., Tran J. B., Krężel A., Galvanization of Protein-Protein Interactions in a Dynamic Zinc Interactome, Trends Biochem. Sci, 2021, 46 (1), 64. 10.1016/j.tibs.2020.08.011 32958327

[45] Jiang L. J., Vašák M., Vallee B., Maret M., Zinc Transfer Potentials of the α- and β-Clusters of Metallothionein Are Affected by Domain Interactions in the Whole Molecule, Proc. Natl. Acad. Sci. U.S.A., 2000, 97 (6), 2503–2508. 10.1073/pnas.97.6.2503 10716985 PMC15958

[46] Drozd A., Wojewska D., Peris-Díaz M. D., Jakimowicz P., Krężel A., Crosstalk of the Structural and Zinc Buffering Properties of Mammalian Metallothionein-2, Metallomics, 2018, 10 (4), 595. 10.1039/C7MT00332C 29561927

[47] Romero-Isart N., Oliva B., Vasák M., Influence of NH-Sgamma Bonding Interactions on the Structure and Dynamics of Metallothioneins, J. Mol. Model., 2010, 16 (3), 387–394. 10.1007/s00894-009-0542-x 19609577

[48] Kochańczyk T., Nowakowski M., Wojewska D., Kocyła A., Ejchart A., Koźmiński W., Krężel A., Metal-Coupled Folding as the Driving Force for the Extreme Stability of Rad50 Zinc Hook Dimer Assembly, Sci Rep, 2016, 6 (1), 36346. 10.1038/srep36346 27808280 PMC5093744

[49] McKnight C. J., Doering D. S., Matsudaira P. T., Kim P. S., A Thermostable 35-Residue Subdomain within Villin Feadpiece, J. Mol. Biol., 1996, 260 (2), ), 126–134. 10.1006/jmbi.1996.0387 8764395

[50] McKnight C. J., Matsudaira P. T., Kim P. S., NMR Structure of the 35-Residue Villin Headpiece Subdomain, Nat. Struct. Mol. Biol., 1997, 4 (3), 180–184. 10.1038/nsb0397-180 9164455

[51] Hong S., Toyama M., Maret W., Murooka Y., High Yield Expression and Single Step Purification of Human Thionein/Metallothionein, Protein Expression Purif., 2001, 21 (1), 243. 10.1006/prep.2000.1372 11162412

[52] Pomorski A., Adamczyk J., Bishop A. C., Krężel A., Probing the Target-Specific Inhibition of Sensitized Protein Tyrosine Phosphatases with Biarsenical Probes, Org. Biomol. Chem., 2015, 13 (5), 1395–1403. 10.1039/C4OB02256D 25460004 PMC4303503

[53] Pomorski A., Krężel A., Biarsenical Fluorescent Probes for Multifunctional Site-Specific Modification of Proteins Applicable in Life Sciences: an Overview and Future Outlook, Metallomics, 2020, 12 (8), 1179–1207. 10.1039/d0mt00093k 32658234

[54] Haase H., Maret W., Partial Oxidation and Oxidative Polymerization of Metallothionein, Electrophoresis, 2008, 29 (20), 4169. 10.1002/elps.200700922 18844317

[55] Krężel A., Latajka R., Bujacz G. D., Bal W., Coordination Properties of Tris(2-Carboxyethyl)Phosphine, a Newly Introduced Thiol Reductant, and Its Oxide, Inorg. Chem., 2003, 42 (6), 1994. 10.1021/ic025969y 12639134

[56] Krężel A., Leśniak W., Jeżowska-Bojczuk M., Młynarz P., Brasuń J., Kozłowski H., Bal W., Coordination of Heavy Metals by Dithiothreitol, a Commonly Used Thiol Group Protectant, J. Inorg. Biochem., 2001, 84 (1–2), 77. 10.1016/S0162-0134(00)00212-9 11330484

[57] Eyer P., Worek F., Kiderlen D., Sinko G., Stuglin A., Simeon-Rudolf V., Reiner E., Molar Absorption Coefficients for the Reduced Ellman Reagent: Reassessment, Anal. Biochem., 2003, 312 (2), 224. 10.1016/S0003-2697(02)00506-7 12531209

[58] Kocyła A., Pomorski A., Krężel A., Molar Absorption Coefficients and Stability Constants of Metal Complexes of 4-(2-pyridylazo)resorcinol (PAR), Revisiting Common Chelating Probe for the Study of Metalloproteins, J. Inorg. Biochem., 2015, 152, 82–92. 10.1016/j.jinorgbio.2015.08.024 26364130

[59] Calvo J. S., Lopez V. M., Meloni G., Non-Coordinative Metal Selectivity Bias in Human Metallothioneins Metal-Thiolate Clusters, Metallomics, 2018, 10 (12), 1777. 10.1039/C8MT00264A 30420986 PMC6450656

[60] Kluska K., Adamczyk J., Krężel A., Metal Binding Properties, Stability and Reactivity of Zinc Fingers, Coord. Chem. Rev., 2018, 367, 18. 10.1016/j.ccr.2018.04.009

[61] Peroza E. A., dos Santos Cabral A., Wan X., Freisinger E.., Metal Ion Release froms Metallothioneins: Proteolysis as an Alternative to Oxidation, Metallomics, 2013, 5 (9), 1204. 10.1039/c3mt00079f 23835914

[62] Pomorski A., Drozd A., Kocyła A., Krężel A., From Methodological Limitations to the Function of Metallothioneins – a Guide to Approaches for Determining Weak, Moderate, and Tight Affinity Zinc Sites, Metallomics, 2023, 10.1093/mtomcs/mfad027 PMC10210066 37113075

[63] Marszałek I., Krężel A., Goch W., Zhukov I., Paczkowska I., Bal W., From Methodological Limitations to the Function of Metallothioneins – a Guide to Approaches for Determining Weak, Moderate, and Tight Affinity Zinc Sites, J. Inorg. Biochem., 2016, 161, 107. 10.1016/j.jinorgbio.2016.05.009 27216451

[64] Hirano T., Kikuchi K., Urano Y., Nagano T., Improvement and Biological Applications of Fluorescent Probes for Zinc, ZnAFs, J. Am. Chem. Soc., 2002, 124 (23), 6555. 10.1021/ja025567p 12047174

[65] Li T. Y., Minkel D. T., Shaw C. F. 3rd, Petering D. H, On the Reactivity of Metallothioneins with 5,5'-Dithiobis-(2-Nitrobenzoic Acid), Biochem. J., 1981, 193 (2), 441. 10.1042/bj1930441 7305942 PMC1162625

[66] Jacob C., Maret W., Vallee B. L., Control of Zinc Transfer between Thionein, Metallothionein, and Zinc Proteins, Proc. Natl. Acad. Sci. U.S.A., 1998, 95 (7), 3489. 10.1073/pnas.95.7.3489 9520393 PMC19863

[67] Yu W. H., Cai B., Gao Y., Xie Y., Huang Z. X., Expression, Characterization, and Reaction of Recombinant Monkey Metallothionein-1 and Its C33M Mutant, J. Protein Chem., 2002, 21 (2), 177–185. 10.1023/A:1015324717115 12018619

[68] Cismowski M. J., Huang P. C., Effect of Cysteine Replacements at Positions 13 and 50 on Metallothionein Structure, Biochemistry, 1991, 30 (26), 6626. 10.1021/bi00240a036 2054361

[69] Cai B., Zheng Q., Teng X. C., Chen D., Wang Y., Wang K. Q., Zhou G. M., Xie Y., Zhang M. J., Sun H., Huang Z. X., The Role of Thr5 in Human Neuron Growth Inhibitory Factor, J. Biol. Inorg. Chem., 2006, 11 (4), 476. 10.1007/s00775-006-0097-6 16601975

[70] Ding Z. C., Teng X. C., Cai B., Wang H., Zheng Q., Wang Y., Zhou G. M., Zhang M. J., Wu H. M., Sun H. Z., Huang Z. X., Mutation at Glu23 Eliminates the Neuron Growth Inhibitory Activity of Human Metallothionein-3, Biochem. Biophys. Res. Commun., 2006, 349 (2), 674–682. 10.1016/j.bbrc.2006.08.090 16945328

[71] Cody C. W., Huang P. C., Metallothionein Detoxification Function Is Impaired by Replacement of both Conserved Lysines with Glutamines in the Hinge between the Two Domains, Biochemistry, 1993, 32 (19), 5127. 10.1021/bi00070a022 8494889

[72] Ding Z. C., Teng X. C., Zheng Q., Ni F. Y., Cai B., Wang Y., Zhou G. M., Sun H. Z., Tan X. S., Huang Z. X., Important Roles of the Conserved Linker-KKS in Human Neuronal Growth Inhibitory Factor, Biometals, 2009, 22 (5), 817–826. 10.1007/s10534-009-9228-1 19306065

[73] Ding Z. C., Zheng Q., Cai B., Yu W. H., Teng X. C., Wang Y., Zhou G. M., Wu H. M., Sun H. Z., Zhang M. J., Huang Z. X., Effect of α-Domain Substitution on the Structure, Property and Function of Human Neuronal Growth Inhibitory Factor, J. Biol. Inorg. Chem., 2007, 12 (8), 1173–1179. 10.1007/s00775-007-0287-x 17712581

[74] Muñoz A., Laib F., Petering D. H., Shaw C. F. 3rd., Characterization of the Cadmium Complex of Peptide 49-61: a Putative Nucleation Center for Cadmium-Induced Folding in Rabbit Liver Metallothionein IIA, J. Biol. Inorg. Chem., 1999, 4 (4), 495. 10.1007/s007750050335 10555583

[75] Reddi A. R., Guzman T. R., Breece R. M., Tierney D. L., Gibney B. R., Deducing the Energetic Cost of Protein Folding in Zinc Finger Proteins Using Designed Metallopeptides, J. Am. Chem. Soc., 2007, 129 (42), 12815–12827. 10.1021/ja073902+ 17902663

[76] Gan T, Munoz A, Shaw C. F. 3rd, Petering D. H., Reaction of ^111^Cd_7_-Metallothionein with EDTA. A Reappraisal, J. Biol. Chem., 1995, 270 (10), 5339. 10.1074/jbc.270.10.5339 7890646

[77] Good M., Hollenstein R., Sadler P. J., Vašák M., ^113^Cd NMR Studies on Metal-Thiolate Cluster Formation in Rabbit Cd(II)-Metallothionein: Evidence for a pH Dependence, Biochemistry, 1988, 27 (18), 7163. 10.1021/bi00418a074 3196709

[78] Vazquez F., Vašák M., Comparative ^113^Cd-N.M.R. Studies on Rabbit ^113^Cd_7_-, (Zn_1_,Cd_6_)- and Partially Metal-Depleted ^113^Cd_6_-Metallothionein-2A, Biochem. J., 1988, 253 (2), 611. 10.1042/bj2530611 3178730 PMC1149342

[79] Irvine G. W., Pinter T. B., Stillman M. J., Defining the Metal Binding Pathways of Human Metallothionein 1A: Balancing Zinc Availability and Cadmium Seclusion, Metallomics, 2016, 8 (1), 71. 10.1039/C5MT00225G 26583802

[80] Kambe T., Yamaguchi-Iwai T. Y., Sasaki R., Nagao M., Overview of Mammalian Zinc Transporters, Cell. Mol. Life Sci., 2004, 61 (1), 49. 10.1007/s00018-003-3148-y 14704853 PMC11138893

[81] Hogstrand C., Fu D. in Binding, Transport and Storage of Metal Ions in Biological Cells, eds. Maret W, Wedd A 2014 (Cambridge, UK: Royal Society of Chemistry), 938.

[82] Clemens S. , The Cell Biology of Zinc, J. Exp. Bot., 2022, 73 (6), 1688. 10.1093/jxb/erab481 34727160

[83] Mehlenbacher M. R., Elsiesy R., Lakha R., Villones R. L. E., Orman M., Vizcarra C. L., Meloni G., Wilcox D. E., Austin R. N., Metal Binding and Interdomain Thermodynamics of Mammalian Metallothionein-3: Enthalpically Favoured Cu^+^ Supplants Entropically Favoured Zn^2+^ to Form Cu_4_^+^ Clusters under Physiological Conditions, Chem. Sci., 2022, 13 (18), 5289. 10.1039/D2SC00676F 35655557 PMC9093145

[84] Weiss A, Murdoch C. C, Edmonds K. A., Jordan M. R., Monteith A. J., Perera Y. R., Rodríguez Nassif A. M., Petoletti A. M., Beavers W. N., Munneke M. J., Drury S. L., Krystofiak W. S., Thalluri K., Wu H., Kruse A. R. S., DiMarchi R. D., Caprioli R. M., Spraggins J. M., Chazin W. J., Giedroc D. P., Skaar E. P., Zn-Regulated GTPase Metalloprotein Activator 1 Modulates Vertebrate Zinc Homeostasis, Cell, 2022, 185 (12), 2148. 10.1016/j.cell.2022.04.011 35584702 PMC9189065

[85] Maret W. , Escort Proteins for Cellular Zinc Ions, Nature, 2022, 608 (7921), 38. 10.1038/d41586-022-01988-2 35918525

[86] Carter K. P., Young A. M., Palmer A. E., Fluorescent Sensors for Measuring Metal Ions in Living Systems, Chem. Rev., 2014, 114 (8), 4564. 10.1021/cr400546e 24588137 PMC4096685

[87] Domaille D. W., Que E. L., Chang C. J., Synthetic Fluorescent Sensors for Studying the Cell Biology of Metals, Nat. Chem Biol, 2008, 4 (3), 168. 10.1038/nchembio.69 18277978

[88] Krężel A., Maret W., Zinc-Buffering Capacity of a Eukaryotic Cell at Physiological pZn, J. Biol. Inorg. Chem., 2006, 11 (8), 1049–1062. 10.1007/s00775-006-0150-5 16924557

[89] Maret W. , Analyzing Free Zinc(II) Ion Concentrations in Cell Biology with Fluorescent Chelating Molecules, Metallomics, 2015, 7 (2), 202. 10.1039/C4MT00230J 25362967

[90] Sensi S. L., Ton-That D., Weiss J. H., Rothe A., Gee K. R., A New Mitochondrial Fluorescent Zinc Sensor, Cell Calcium, 2003, 34 (3), 281. 10.1016/S0143-4160(03)00122-2 12887975

[91] Zaia J., Jiang L., Han M. S., Tabb J. R., Wu Z., Fabris D., Fenselau C. A., A Binding Site for Chlorambucil on Metallothionein, Biochemistry, 1996, 35 (9), 2830. 10.1021/bi952243n 8608118

[92] Fan L., Russell D. H., An Ion Mobility-Mass Spectrometry Study of Copper-Metallothionein-2A: Binding Sites and Stabilities of Cu-MT and Mixed Metal Cu-Ag and Cu-Cd Complexes, Analyst, 2023, 148 (3), 546. 10.1039/D2AN01556K 36545796 PMC9904198

[93] Ejnik J., Robinson J., Zhu J., Försterling H., Shaw C. F. 3rd, Petering D. H., Folding Pathway of Apo-Metallothionein Induced by Zn^2+^, Cd^2+^ and Co^2+^, J. Inorg. Biochem., 2002, 88 (2), 144. 10.1016/S0162-0134(01)00393-2 11803035

[94] Bertini I., Luchinat C., Messori L., Vasak M., Proton NMR Studies of the Cobalt(II)-Metallothionein System, J. Am. Chem. Soc., 1989, 111 (19), 7296–7300. 10.1021/ja00201a002

[95] Adams S. R., Campbell R. E., Gross L. A., Martin B. R., Walkup G. K., Yao Y., Llopis J., Tsien R. Y., New Biarsenical Ligands and Tetracysteine Botifs for Protein Labeling *In Vitro* and *In Vivo*: Synthesis and Biological Applications, J. Am. Chem. Soc., 2002, 124 (21), 6063–6076. 10.1021/ja017687n 12022841

[96] Macchiagodena M., Pagliai M., Andreini C., Rosato A., Procacci P., Upgrading and Validation of the AMBER Force Field for Histidine and Cysteine Zinc(II)-Binding Residues in Sites with Four Protein Ligands, J. Chem. Inf. Model., 2019, 59 (9), 3803–3816. 10.1021/acs.jcim.9b00407 31385702

[97] Kluska K., Chorążewska A., Peris-Díaz M. D., Adamczyk J., Krężel A., Non-Conserved Amino Acid Residues Modulate the Thermodynamics of Zn(II) Binding to Classical ββα Zinc Finger Domains, Int. J. Mol. Sci., 2022, 23 (23), 14602. 10.3390/ijms232314602 36498928 PMC9735795

[98] Van Der Spoel D., Lindahl E., Hess B., Groenhof G., Mark A. E., Berendsen H. J. C., GROMACS: Fast, Flexible, and Free, J. Comput. Chem., 2005, 26 (16), 1701–1718. 10.1002/jcc.20291 16211538

[99] PLUMED consortium ., Promoting Transparency and Reproducibility in Enhanced Molecular Simulations, Nat. Methods, 2019, 16 (8), 670. 10.1038/s41592-019-0506-8 31363226

