# Supplementary material for: The connection of α- and β-domains in mammalian metallothionein-2 differentiates Zn(II) binding affinities, affects folding, and determines zinc buffering properties
Source: Metallomics. 2023 May 5;15(6):mfad029. doi: 10.1093/mtomcs/mfad029 (PMC10243857; doi:10.1093/mtomcs/mfad029)
Supplement: mfad029_Supplemental_File [file mfad029_supplemental_file.docx]

*Supplementary data*

The connection of α- and β-domains in mammalian metallothionein-2 differentiates Zn(II) binding affinities, affects folding, and determines zinc buffering properties

Avinash Kumar Singh,*^#^* Adam Pomorski,^#^ Sylwia Wu,^#^ Manuel D. Peris-Díaz , Hanna Czepczyńska-Krężel and Artur Krężel*

*Contribution from*

*Department of Chemical Biology, Faculty of Biotechnology, University of Wrocław, Joliot-Curie 14A, 50-383 Wrocław, Poland*

*^#^*These authors contributed equally

*To whom correspondence should be addressed

**Keywords**: metal binding affinity; zinc-sulfur clusters, free zinc, protein thermodynamics, fluorescent probe

**Table S1**. Amino acid sequences of all MT2 protein, its mutants, and domains used in this study. Residues highlighted in the yellow show either mutated positions or added HP35 fragment of villin-1 protein.

| Protein name | Amino acid sequence |
| --- | --- |
| MT2 (WT) | MDPNCSCAAGDSCTCAGSCKCKECKCTSCKKSCCSCCPVGCAKCAQGCICKGASDKCSCCA |
| αMT2 | KKSCCSCCPVGCAKCAQGCICKGASDKCSCCA |
| βMT2 | MDPNCSCAAGDSCTCAGSCKCKECKCTSCKKS |
| S6A-MT2 | MDPNC**A**CAAGDSCTCAGSCKCKECKCTSCKKSCCSCCPVGCAKCAQGCICKGASDKCSCCA |
| K20A-MT2 | MDPNCSCAAGDSCTCAGSC**A**CKECKCTSCKKSCCSCCPVGCAKCAQGCICKGASDKCSCCA |
| C21A-MT2 | MDPNCSCAAGDSCTCAGSCK**A**KECKCTSCKKSCCSCCPVGCAKCAQGCICKGASDKCSCCA |
| K30A-MT2 | MDPNCSCAAGDSCTCAGSCKCKECKCTSC**A**KSCCSCCPVGCAKCAQGCICKGASDKCSCCA |
| K31A-MT2 | MDPNCSCAAGDSCTCAGSCKCKECKCTSCK**A**SCCSCCPVGCAKCAQGCICKGASDKCSCCA |
| K30A_K31A-MT2 | MDPNCSCAAGDSCTCAGSCKCKECKCTSC**AA**SCCSCCPVGCAKCAQGCICKGASDKCSCCA |
| βMT2-villin | MDPNCSCAAGDSCTCAGSCKCKECKCTSCKKSLSDEDFKAVFGMTRSAFANLPLWLQQHLLKEKGLF |

**Table S2**. Expected and observed molecular masses of MT2, its domains, and mutants. Average molecular masses were calculated using Compute pI/Mw tool from the Expasy server.^1^

| Protein | Mass calculated (Da) | Mass observed (Da) |
| --- | --- | --- |
| MT2 (WT) | 6042.16 | 6042.2 |
| α-domain (αMT2) | 3149.82 | 3149.4 |
| β-domain (βMT2) | 3253.78 | 3253.2 |
| S6A-MT2 | 6026.17 | 6026.3 |
| K20A-MT2 | 5985.07 | 5985.3 |
| C21A-MT2 | 6010.1 | 6009.4 |
| K30A-MT2 | 5985.07 | 5984.4 |
| K31A-MT2 | 5985.07 | 5986.3 |
| K30A,K31A-MT2 | 5927.97 | 5927.2 |
| βMT2-villin | 7287.49 | 7286.9 |

**Mutagenesis of the MT2a sequence**

The following point mutations of human MT2A (K30A-MT2, K31A-MT2, K30AK31A-MT2) have been performed using Agilent QuickChange Site-directed mutagenesis kit protocol.^2^ Briefly: primers complementary to each other and template DNA were designed. The primers sequences are:

For K30A-MT2 5’ GCACCAGCTGCGCAAAAAGCTGCTGCAGC 3’

Rev K30A-MT2 5’ GCTGCAGCAGCTTTTTGCGCAGCTGGTGC 3’

For K31A-MT2 5’ GCACCAGCTGCAAAGCAAGCTGCTGCAGC 3’

Rev K31A-MT2 5’ GCTGCAGCAGCTTGCTTTGCAGCTGGTGC 3’

For K30AK31A-MT2 5’ GCACCAGCTGCGCAGCAAGCTGCTGCAGC 3’

Rev K30AK31A-MT2 5’ GCTGCAGCAGCTTGCTGCGCAGCTGGTGC 3’

PCR program:

1-st step 95°C for 30 s

2-nd step repeated 16 x

95°C for 30 s

55°C for 1 min

68°C for 1 min/1 kb of template plasmid DNA

PCR reaction volume = 50 µl

To introduce the S6A-MT2, K20A-MT2, and C21A-MT2 mutation, the Quick-Change protocol was also applied, however not successfully, despite multiple attempts. Therefore an improved protocol using primers that are not fully overlapping was utilized,^3^ and designed mutations were obtained.

For MT2A S6A-MT2 5’C AACTGCGCCTGTGCGGCGGGT 3’

Rev MT2A S6A-MT2 5’ CGCACAGGCGCAGTTCGGATCCAT 3’

For K20A-MT2 5’CTGCGCGTGCAAAGAATGCAAATGC 3’

Rev K20A-MT2 5’ TTTGCACGCGCAGCTGCCCG 3’

For C21A-MT2 5’ GCTGCAAAGCGAAAGAATGCAAATGCAC 3’

Rev C21A-MT2 5’ GCGGGCAGCTGCAAAGCGAAAGAAT 3’

PCR program:

1-st step 94°C for 60 s

2-nd step repeated 16 x

94°C for 1 min

68°C for 1 min

72°C for 8 min

Final elongation 72°C for 8 min

PCR reaction volume = 25 µl

PCR product has been digested with DpnI enzyme 37°C/4 h to eliminate the template DNA. After digestion, competent *E. coli* cells (DH5α) were transformed with 10 µl of the mixture using the heat-shock method and cultivated on LB agar plates with 150 µg/ml Amp. The plasmid DNA from a single bacterial colony has been isolated using Plasmid Mini Kit (Syngene) and sequenced (Microsynth AG).

**Figure S1.** A representative SDS-PAGE gel from the purification of MT2. Lane 1 is *E.coli* RIL prior to induction. Lane 2 is the precipitate after sonication. The band represents the CBP-intein-MT2 fusion. Lane 3 is the soluble fraction. Lane 4 is flow-through after the chitin column, and 5 is a wash. Lane 6 represents the eluted MT2 that was cleaved from the intein. The smear is characteristic of metallothionein due to oxidative polymerization. The upper panel was stained with Coomassie, and the lower is the fluorescence of the F4-FlAsH moiety bound to MT2.

**Exemplary calculations of dissociation constants of Zn(II)-MT proteins used in this study with 4-(2-pyridylazo)resorcinol (PAR)**

*Calculation of K_d1_ of Zn_3_βMT2 (Zn(II)-loaded β-domain of MT2):*

Dissociation of the weakest Zn(II) ion from Zn_3_βMT2 occurs according to the Eq. 1

Zn_3_βMT2 + 2PAR ⇌ Zn_2_βMT2 + Zn(PAR)_2_  (Eq. 1)

The concentration of Zn_3_βMT2 was 1.7 µM, while PAR was 200 µM in 2 ml HEPES buffer (see Experimental section). Absorbance of Zn(PAR)_2_ complex measured at 492 nm in equilibrium state was 0.0383. Based on its molar absorption coefficient (71,500 M^-1^⋅cm^-1^) concentrations of reactants are:

[Zn(PAR)_2_] = 5.36⋅10^-7^ M (chromogenic complex)

[PAR] = 2⋅10^-4^ - 5.36⋅10^-7^ M = 1.9946⋅10^-4^ M (free PAR)

[Zn_3_βMT2] = Zn_3_βMT2_Total_ - [Zn(PAR)_2_] = 1.7⋅10^-6^ M - 5.36⋅10^-7^ M = 1.16⋅10^-6^ M

[Zn_2_βMT2] = Zn_3_βMT2_Total_ - [Zn_3_βMT2] = 5.36⋅10^-7^ M

Exchange constant *K*_ex1_ is described by Eq. 2

$K_{ex1}=\frac{[Zn\left( \mathrm{PAR} \right)_{2}]\cdot[\mathrm{Zn}_{2}MT2]}{\left[ \mathrm{Zn}_{3}MT2 \right]\cdot{[\mathrm{PAR}]}^{2}}$ (Eq. 2)

The *K*_ex1_ value after substituting the concentrations of reactants is **6.19** **M^-1^**. Finally *K*_d1_ (the first dissociation constant) is calculated using Eq. 3 and known dissociation constant of Zn(PAR)_2_, *K*_d12_^PAR^ = 7.1⋅10^-13^ M^2^.

*K*_d1_ = $\frac{\left[ \mathrm{Zn}_{2}MT2 \right]\left[ \mathrm{Zn}\left( \mathrm{II} \right) \right]_{\mathrm{free}}}{\left[ \mathrm{Zn}_{3}MT2 \right]}=$*K*_ex1_⋅ *K*_d12_^PAR^ (Eq. 3)

*K*_d1_ = 6.19 M^-1^ **⋅** 7.1⋅10^-13^ M^2^ = 4.39⋅10^-12^ M

-log*K*_d1_ = **11.36**

*Calculation of K_d12_^av^ of Zn_7_MT2 (Zn(II)-loaded MT2):*

Dissociation of two weakest Zn(II) ions from MT2 in exchange reaction with PAR occurs according to the Eq. 4.

Zn_7_MT2 + 4PAR ⇌ Zn_5_MT2 + 2Zn(PAR)_2_ (Eq. 4)

The concentration of MT2 was 1.7 µM, while PAR was 200 µM in 2 ml HEPES buffer (see Experimental section). Absorbance of Zn(PAR)_2_ complex measured at 492 nm in equilibrium state was 0.1386. Based on its molar absorption coefficient (71,500 M^-1^⋅cm^-1^)^4^ concentrations of reactants are:

[Zn(PAR)_2_] = 1.94⋅10^-6^ M (chromogenic complex)

[PAR] = 2⋅10^-4^ - 1.94⋅10^-6^ M = 1.9806⋅10^-4^ M (free PAR)

[Zn_7_MT2] = Zn_7_MT2_Total_ - [Zn(PAR)_2_]/2 = 1.7⋅10^-6^ M - 9.69⋅10^-7^ M = 7.31⋅10^-7^ M

Dividing the [Zn(PAR)_2_] concentration by 2 results from Eq. 4.

[Zn_5_MT2] = Zn_3_βMT2_Total_ - [Zn_7_MT2] = 9.69⋅10^-7^ M

Exchange constant *K*_ex12_ is described by Eq. 5

$K_{ex12}=\frac{[\mathrm{Zn}_{5}MT2]{[Zn\left( \mathrm{PAR} \right)_{2}]}^{2}}{\left[ \mathrm{Zn}_{7}MT2 \right]\cdot{[\mathrm{PAR}]}^{4}}$ (Eq. 5)

The *K*_ex12_ value after substituting the concentrations of reactants is **3114** **M^-2^**. Finally *K*_d12_ (cumulative dissociation constant of the first two events) is calculated using Eq. 6 and known dissociation constant of Zn(PAR)_2_, *K*_d12_^PAR^ = 7.1⋅10^-13^ M^2^.^4^

*K*_d12_ = $\frac{\left[ \mathrm{Zn}_{5}MT2 \right]{[\mathrm{Zn}\left( \mathrm{II} \right)]}_{\mathrm{free}}^{2}}{\left[ \mathrm{Zn}_{7}MT2 \right]}$=*K*_ex12_⋅ (*K*_d12_^PAR^)^2^ (Eq. 6)

*K*_d12_ = 3114 M^-2^ ⋅ (7.1⋅10^-13^ M^2^)^2^ = 1.56⋅10^-21^ M^2^

Since *K*_d12_ is a cumulative constant of two events and its direct comparison with *K*_d1_ is impossible, we used here *K*_d12_^av^ being average value of *K*_d1_ and *K*_d2_. *K*_d12_^av^ is obtained by square root of *K*_d12_ according to Eq. 7.

$K_{d12}^{av}=\sqrt{K_{d12}}$ (Eq. 7)

*K*_d12_^av^ = $\sqrt{1.56{10}^{-21} M^{2}}=3.95{10}^{-11}M$

*-*log*K*_d12_^av^ = **10.40**

**Figure S2.** Example measurement of oxidation of MT2, its domains, and mutants (1.7 µM) with 2 µM DTNB in 50 mM HEPES buffer pH 7.4, 0.1 M NaCl.

| Zn(II) site | Frequency ocurrance (%) | Label pathway | Order of the Zn−S bond break | | | | Mean rupture force (kcal/mol) | Total work done (kcal/mol) |
| --- | --- | --- | --- | --- | --- | --- | --- | --- |
|  |  |  | 1 | 2 | 3 | 4 |  |  |
| **IV** | **80** | **A** | **Cys7** | **Cys24** | **Cys21** | **Cys5** | **36 ± 13** | **411 ± 26** |
| IV | 20 | B | Cys7 | Cys21 | Cys24 | Cys5 | 33 ± 13 | 384 ± 5 |
| III | 20 | A | Cys7 | Cys26 | Cys15 | Cys13 | 31 ± 10 | 348 ± 13 |
| **III** | **50** | **B** | **Cys26** | **Cys7** | **Cys15** | **Cys13** | **30 ± 9** | **341 ± 9** |
| **II** | **60** | **A** | **Cys24** | **Cys15** | **Cys29** | **Cys19** | **35 ± 13** | **412 ± 16** |
| II | 40 | B | Cys24 | Cys15 | Cys19 | Cys29 | 34 ± 14 | 397 ± 9 |

**Table S3**. Steered MD simulations for the Zn_7_MT2 system considering all the unbinding pathways.


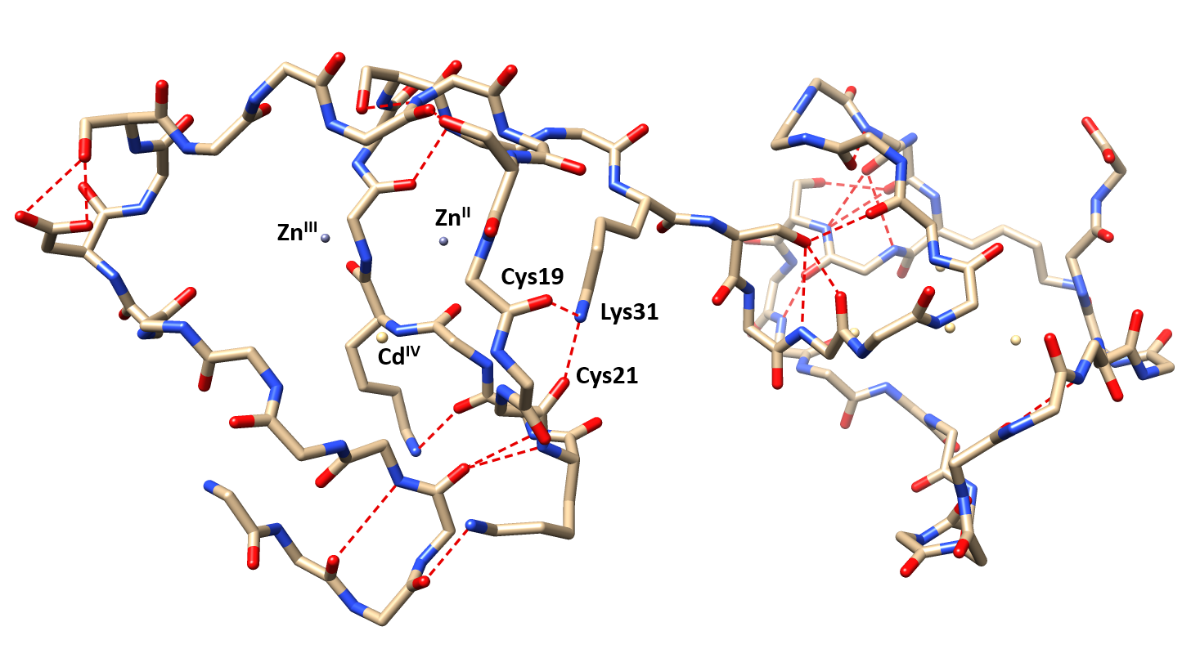


**Figure S3.** Intra- and interdomain hydrogen bonding network (dashed red lines) in the crystal structure of hepatic rat Cd_5_Zn_2_MT2 (PDB: 4MT2).^5^ Two hydrogen bonds between the amine function of Lys31 (α-domain) and carbonyl oxygen atoms from Cys19 and Cys21 (β-domain) are highlighted. Note that other than identified in the X-ray structure, interactions are not presented.

**References:**

1. Expasy ProtParam tool, <https://web.expasy.org/protparam/>
2. J. Braman, C. Papworth and A. Greener, Site-directed mutagenesis using double-stranded plasmid DNA templates. *Methods Mol. Biol.* 1996, **57**, 31−44.
3. Zheng L, Baumann U, Reymond JL. An efficient one-step site-directed and site-saturation mutagenesis protocol. *Nucleic Acids Res.* 2004, **32**: e115.
4. A. Kocyła, A. Pomorski, A. Krężel, Molar absorption coefficients and stability constants of metal complexes of 4-(2-pyridylazo)resorcinol (PAR): Revisiting common chelating probe for the study of metalloproteins. *J. Inorg. Biochem.* 2015, **152**, 82−92.
5. A. H. Robbins, D. E. McRee, M. Williamson, S. A. Collett, N. H. Xuong, W. F. Furey, B. C. Wang and C. D. Stout, *J. Mol. Biol.*, 1991, **221**, 1269–1293.
